# Conserved cobalamin acquisition protein 1 is essential for vitamin B_12_ uptake in both *Chlamydomonas* and *Phaeodactylum*

**DOI:** 10.1093/plphys/kiad564

**Published:** 2023-10-21

**Authors:** Andrew P Sayer, Marcel Llavero-Pasquina, Katrin Geisler, Andre Holzer, Freddy Bunbury, Gonzalo I Mendoza-Ochoa, Andrew D Lawrence, Martin J Warren, Payam Mehrshahi, Alison G Smith

**Affiliations:** Department of Plant Sciences, University of Cambridge, Downing Street, Cambridge CB2 3EA, UK; Department of Plant Sciences, University of Cambridge, Downing Street, Cambridge CB2 3EA, UK; Department of Plant Sciences, University of Cambridge, Downing Street, Cambridge CB2 3EA, UK; Department of Plant Sciences, University of Cambridge, Downing Street, Cambridge CB2 3EA, UK; Department of Plant Sciences, University of Cambridge, Downing Street, Cambridge CB2 3EA, UK; Department of Plant Sciences, University of Cambridge, Downing Street, Cambridge CB2 3EA, UK; School of Biological Sciences, University of Southampton, Southampton SO17 1BJ, UK; School of Biosciences, University of Kent, Canterbury, Kent CT2 7NJ, UK; Quadram Institute Bioscience, Norwich Research Park, Norwich NR4 7UA, UK; Department of Plant Sciences, University of Cambridge, Downing Street, Cambridge CB2 3EA, UK; Department of Plant Sciences, University of Cambridge, Downing Street, Cambridge CB2 3EA, UK

## Abstract

Microalgae play an essential role in global net primary productivity and global biogeochemical cycling. Despite their phototrophic lifestyle, over half of algal species depend for growth on acquiring an external supply of the corrinoid vitamin B_12_ (cobalamin), a micronutrient produced only by a subset of prokaryotic organisms. Previous studies have identified protein components involved in vitamin B_12_ uptake in bacterial species and humans. However, little is known about its uptake in algae. Here, we demonstrate the essential role of a protein, cobalamin acquisition protein 1 (CBA1), in B_12_ uptake in *Phaeodactylum tricornutum* using CRISPR-Cas9 to generate targeted knockouts and in *Chlamydomonas reinhardtii* by insertional mutagenesis. In both cases, CBA1 knockout lines could not take up exogenous vitamin B_12_. Complementation of the *C. reinhardtii* mutants with the wild-type *CBA1* gene restored B_12_ uptake, and regulation of *CBA1* expression via a riboswitch element enabled control of the phenotype. When visualized by confocal microscopy, a YFP-fusion with *C. reinhardtii* CBA1 showed association with membranes. Bioinformatics analysis found that CBA1-like sequences are present in all major eukaryotic phyla. In algal taxa, the majority that encoded CBA1 also had genes for B_12_-dependent enzymes, suggesting CBA1 plays a conserved role. Our results thus provide insight into the molecular basis of algal B_12_ acquisition, a process that likely underpins many interactions in aquatic microbial communities.

## Teaser

Knockout mutants and physiological studies demonstrate that vitamin B_12_ uptake in both *Chlamydomonas reinhardtii* and the unrelated *Phaeodactylum tricornutum* requires cobalamin acquisition protein 1.

## Introduction

Microalgae are a diverse group of eukaryotic organisms that thrive in all aquatic environments. They form the basis of most aquatic food chains and are major contributors to global primary productivity, with marine microalgae responsible for an estimated 30% of total carbon fixation (Field et al. 1998). Understanding the drivers that support algal growth is thus of considerable ecological importance. Despite their photoautotrophic lifestyle, a widespread trait in algae is dependence on an external source of an organic micronutrient, vitamin B_12_ (cobalamin), a complex cobalt-containing corrinoid molecule. Approximately half of algal species surveyed across the eukaryotic tree of life require B_12_ for growth (Croft et al. 2005). However, the proportion of B_12_-dependent species differs between algal groups, from 30% (*n* = 148) of Chlorophytes to 96% (*n* = 27) of algal species that participate in harmful algal blooms (Tang et al. 2010). Within algal lineages, there is no evidence that any can produce B_12_ de novo, so this auxotrophy is not due to loss of one or more biosynthetic genes. Rather, the requirement for B_12_ stems from the fact that it is an essential cofactor for methionine synthase (METH), and species that can grow without supplementation have an alternative, B_12_-independent, isoform of this enzyme called METE (Croft et al. 2005; Helliwell et al. 2011). Many microalgae, including the green alga *Chlamydomonas reinhardtii* and the unrelated diatom *Phaeodactylum tricornutum*, encode both forms of METH and utilize METE in the absence of exogenous B_12_, but take up and utilize the compound if it becomes available (Helliwell et al. 2011). Under those conditions, the expression of *METE*, which has been found to have a lower catalytic rate than METH (Gonzalez et al. 1992), is repressed, and cells rely on METH activity.

The biosynthetic pathway for B_12_ is confined to prokaryotes (Warren et al. 2002) and indeed only a subset of bacteria encode the entire set of 20 or so enzymes required to synthesize corrinoids from the common tetrapyrrole precursor (Shelton et al. 2019), with many eubacterial species also reliant on an external source. In some cases, this is due to the loss of one or a few enzymes of the biosynthetic pathway, but in many bacteria the pathway is absent altogether and auxotrophy is the consequence of relying on one or more B_12_-dependent enzymes, such as METH. In microalgae, supplementation of cultures of *P. tricornutum* with B_12_ increases its growth rate subtly (Bertrand et al. 2012) and in *C. reinhardtii* use of METH confers thermal tolerance (Xie et al. 2013). More direct evidence for a selective advantage is demonstrated by the fact that an experimentally evolved *metE* mutant of *C. reinhardtii* predominates in mixed populations with WT cells over tens of cell generations, as long as B_12_ is included in the medium (Helliwell et al. 2015). This is despite the fact that in the absence of B_12_, the *metE* mutant is nonviable within a few days (Bunbury et al. 2020).

The minimum levels of B_12_ in the medium needed to support growth of laboratory cultures of algal B_12_-auxotrophs are in the range of 10 to 50 pM (Croft et al. 2005), whereas B_12_ concentrations have been reported to be just 5 to 13 pM in freshwater systems (Ohwada 1973). A similar value of 6.2 pM is the average value in most marine environments, although up to 87 pM could be detected in some coastal waters (Sañudo-Wilhelmy et al. 2014), which may be linked to the higher cobalt concentrations measured there (Panzeca et al. 2009). Given the limiting levels of B_12_ in the environment, its relatively short half-life (in the order of days) in surface water (Carlucci et al. 2007; Sañudo-Wilhelmy et al. 2014), and that as a large polar molecule it is unlikely to simply diffuse across cellular membranes, it is clear that algae must have an efficient means to take up B_12_. In bacteria, the molecular mechanisms for B_12_ uptake have been extensively characterized. The B_12_ transport and utilization (*btu*) operon is perhaps the best known (Kadner 1990), comprising BtuB, a TonB-dependent transporter in the outer membrane, a B_12_-binding protein, BtuF, located in the periplasm, and BtuC and BtuD, components of an ATP-binding cassette (ABC) transporter that sits in the inner membrane (Borths et al. 2002). In mammals, dietary B_12_ is bound to intrinsic factor in the ileum and taken up from the gut via receptor-mediated endocytosis (Nielsen et al. 2012). It is then transported between and within cells via multiple B_12_ transport proteins (Banerjee et al. 2021; Choi and Ford 2021). These include lipocalin-1 interacting membrane receptor domain-containing protein 1 (LMBD1), ATP-binding cassette subfamily D member 4 (ABCD4), the latter being an integral membrane ABC transporter in the lysosomal membrane of gut epithelial cells, which facilitates delivery of B_12_ into the cytosol, and multidrug resistant protein 1 (MRP1, also known as ABCC1), another ABC transporter that has sequence similarity to BtuCD and is involved in export of free B_12_ into the plasma where it binds to the main B_12_ transport protein, transcobalamin (Beedholm-Ebsen et al. 2010). Mice *mrp1* mutants were still able to transport a small amount of cobalamin out of cells, indicating redundant mechanisms for this function that have not yet been identified. Cobalamin circulating in the plasma bound to transcobalamin can then be taken up by other cells via receptor-mediated endocytosis (Nielsen et al. 2012).

In contrast to these well-studied processes in bacteria and mammals, the understanding of B_12_ acquisition in microalgae is more limited. A survey of microalgal species, including marine and freshwater taxa and those that require B_12_ (e.g. *Euglena gracilis*, *Thalassiosira pseudonana*) and nonrequirers (such as *P. tricornutum*, *Dunaliella primolecta*), found that many released a “B_12_-binder” into the medium, likely a protein, that appeared to sequester B_12_ from solution and thereby inhibited growth of B_12_-dependent algae (Pintner and Altmeyer 1979). Its role was unknown, but it was postulated that it might be involved in competition for resources between microalgal species in the environment. Subsequently, a protein was purified from the medium of cultures of *T. pseudonana* with a high affinity binding constant of 2 pM for B_12_ (Sahni et al. 2001). In its native state it was an oligomer of >400 kDa, with subunits of ∼80 kDa and the amino acid profile was determined, but it was not possible to obtain sufficient amounts to characterize further. A different approach was taken by Bertrand et al. (2012), who conducted a transcriptomics and proteomics study of *P. tricornutum* and *T. pseudonana* grown under low or sufficient B_12_ conditions. This led to the identification of a gene highly upregulated at the transcript and protein level in the absence of B_12_. Overexpression of this protein in *P. tricornutum* resulted in an increase in the rate of B_12_ uptake, and the protein was named cobalamin acquisition protein 1 (CBA1) although no direct role was established. In this study, we have taken a mutagenesis approach to identify genes responsible for B_12_ uptake in both *P. tricornutum* and *C. reinhardtii*, including extending the work on CBA1. In addition, we have determined the extent to which candidate proteins are conserved throughout the algal lineages, making use of recent increases in algal sequencing data.

## Results

### *P. tricornutum CBA1* knockout lines do not take up B_12_

**Figure 1.**
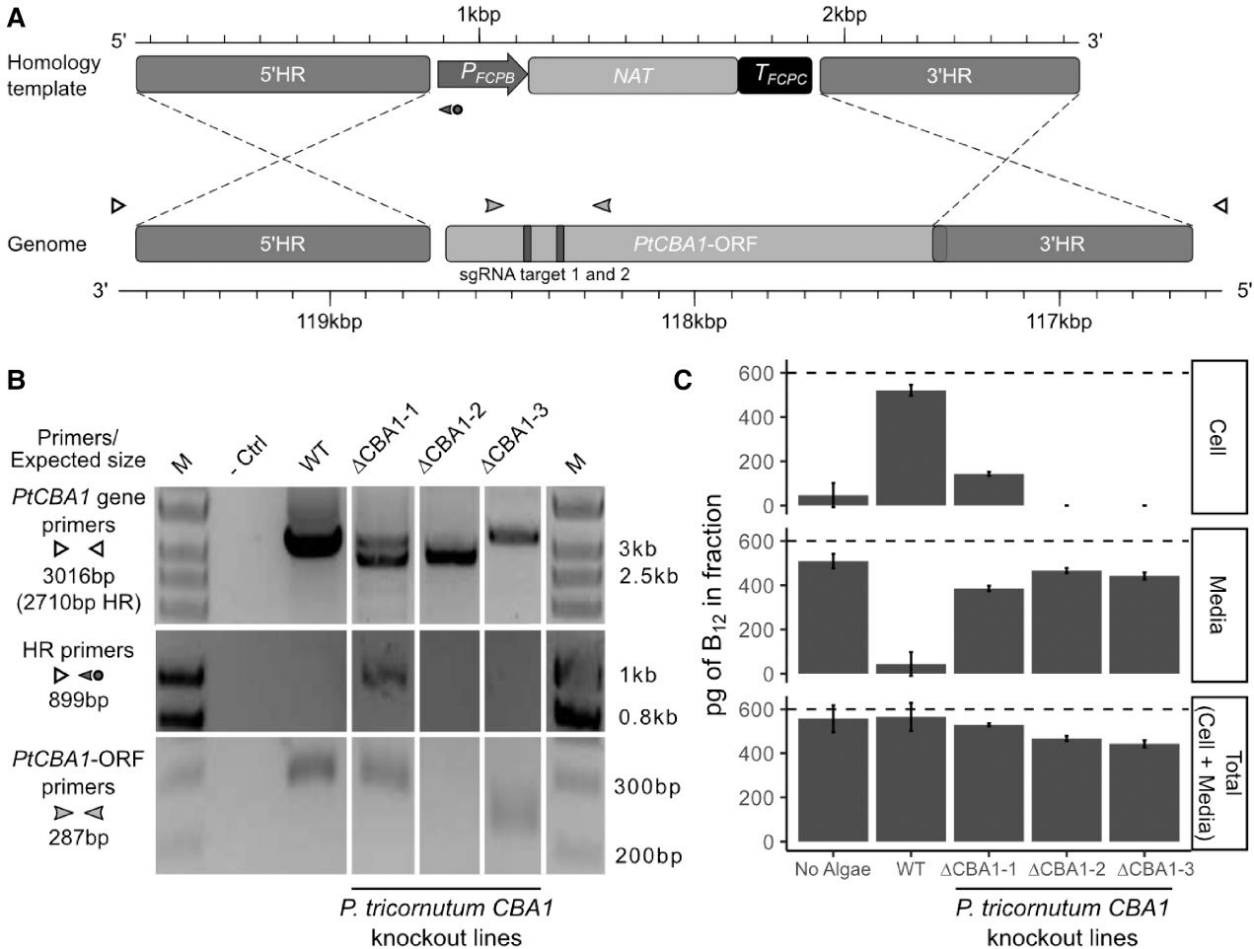
Disruption of *P. tricornutum CBA1* (*PtCBA1*) using CRISPR-Cas9 yielded lines with impaired B_12_ uptake. **A)** Schematic showing CRISPR-Cas9 sgRNA target sites and the homology repair template design used to generate mutant lines in *PtCBA1* (Phatr3_J48322). The homology repair template schematic is annotated with the 5′ homology region (HR) and 3′HR, the *FCPB* promoter, *NAT*, and *FCPC* terminator. The *PtCBA1* gene is annotated with the ORF, the 5′HR and 3′HR regions used in the homology template and the regions of the ORF targeted by sgRNA (vertical bars). Primer positions used for the analysis of putative mutant lines are shown with arrowheads. **B)** PCR of regions across and within WT and mutant *PtCBA1* in three independent CRISPR-Cas9 lines (ΔCBA1) showing indel mutations in the mutants. PCR products from different sets of primers indicated in panel A are shown. M = marker, -Ctrl = no DNA template. **C)** A B_12_ uptake assay was performed as described in the section Materials and Methods, to determine the amount of B_12_ in the media and the cells after 1 h incubation of *P. tricornutum* cells in 600 pg B_12_. The “Total” was inferred by the addition of the cell and media fractions. The dashed line indicates the amount of B_12_ added to the experiment. Standard deviation error bars are shown, *n* = 4. Statistical analysis was performed on the media fraction, and Tukey's test identified the following comparisons to be significantly different from one another: WT vs No Algae (*P* < 1e^−12^); WT vs ΔCBA1-1 (*P* < 1e^−10^); WT vs ΔCBA1-2 (*P* < 1e^−12^); WT vs ΔCBA1-3 (*P* < 1e^−11^); No Algae vs ΔCBA1-1 (*P* < 1e^−03^); No Algae vs ΔCBA1-3 (*P* < 0.05); and ΔCBA1-1 vs ΔCBA1-2 (*P* < 1e^−02^).

Previous work showed that overexpression of CBA1 in *P. tricornutum* conferred enhanced B_12_ uptake rates (Bertrand et al. 2012) but the study did not demonstrate whether it was essential for this process. To address this question, *CBA1* knockout lines were generated in *P. tricornutum* strain 1055/1 (Supplemental Table S1) by CRISPR-Cas9 editing, using a homologous recombination repair template that included a nourseothricin resistance (*NAT*) cassette (Fig. 1A). CRISPR-Cas9 lines were cultured on selective media and screened for the absence of WT alleles at the *PtCBA1* locus (Phatr3_J48322) using PCR (Fig. 1B). When the *PtCBA1* gene was amplified (top panel, Fig. 1B) from ΔCBA1-1 with primers flanking the homologous recombination regions, two bands were detected; the larger of these corresponded to the WT amplicon, whilst the smaller band corresponded to a replacement of *CBA1* by *NAT,* suggesting that this strain is a mono-allelic knockout. For ΔCBA1-2, the *PtCBA1* gene primers amplified a single smaller product, suggesting that this was a bi-allelic knockout, whereas the *PtCBA1* ORF primers (bottom panel, Fig. 1B) did not amplify anything, indicating a disruption specifically in this region. Similarly, no band was detected with primers that amplify across the 5′ end of the NAT knock-in (homology region (HR) primers), which might indicate further disruptions upstream of the 5′HR region of ΔCBA1-2. Although a larger band than for WT was amplified in ΔCBA1-3 using the *PtCBA1* gene primers, those for the *PtCBA1* ORF amplified a smaller product; in both cases a single band was observed indicating a bi-allelic deletion at the sgRNA target sites.

To test whether the ΔCBA1 lines were affected in their ability to take up vitamin B_12_ we developed a standardized B_12_-uptake assay, detailed in the section Materials and Methods. In brief, algal cells were grown to the same growth stage and adjusted to the same cell density, then incubated in media containing a known amount of cyanocobalamin for 1 h. Thereafter, cells were pelleted by centrifugation and the amount of B_12_ determined in the cell pellet and the media fraction using a *Salmonella typhimurium* bioassay (Bunbury et al. 2020). For each sample, the B_12_ measured in the cellular and media fractions were added to provide an estimated “Total” and compared to the amount of B_12_ added initially (Fig. 1C, dashed line), to determine the extent of recovery. For the WT strain, most of the added B_12_ was found in the cellular fraction. The mono-allelic knockout line ΔCBA1-1 consistently showed ∼20% to 30% B_12_ uptake relative to the WT strain. This suggested that a single copy of *PtCBA1* is sufficient to confer B_12_ uptake in *P. tricornutum*, but not to the same extent as the WT strain. In contrast, for the two bi-allelic knockout lines (ΔCBA1-2 and ΔCBA1-3) no B_12_ was detected in the cellular fraction in any experiment, indicating that vitamin B_12_ uptake was fully impaired in the absence of a functional *PtCBA1* copy, at least at the limit of detection of the B_12_ bioassay (of the order of 10 pg). These results expand our understanding of *PtCBA1* by demonstrating that its presence is essential for B_12_ uptake and indicates that there is no functional redundancy to *PtCBA1*.

### Insertional mutagenesis identified the *C. reinhardtii* homologue of *CBA1*

**Figure 2.**
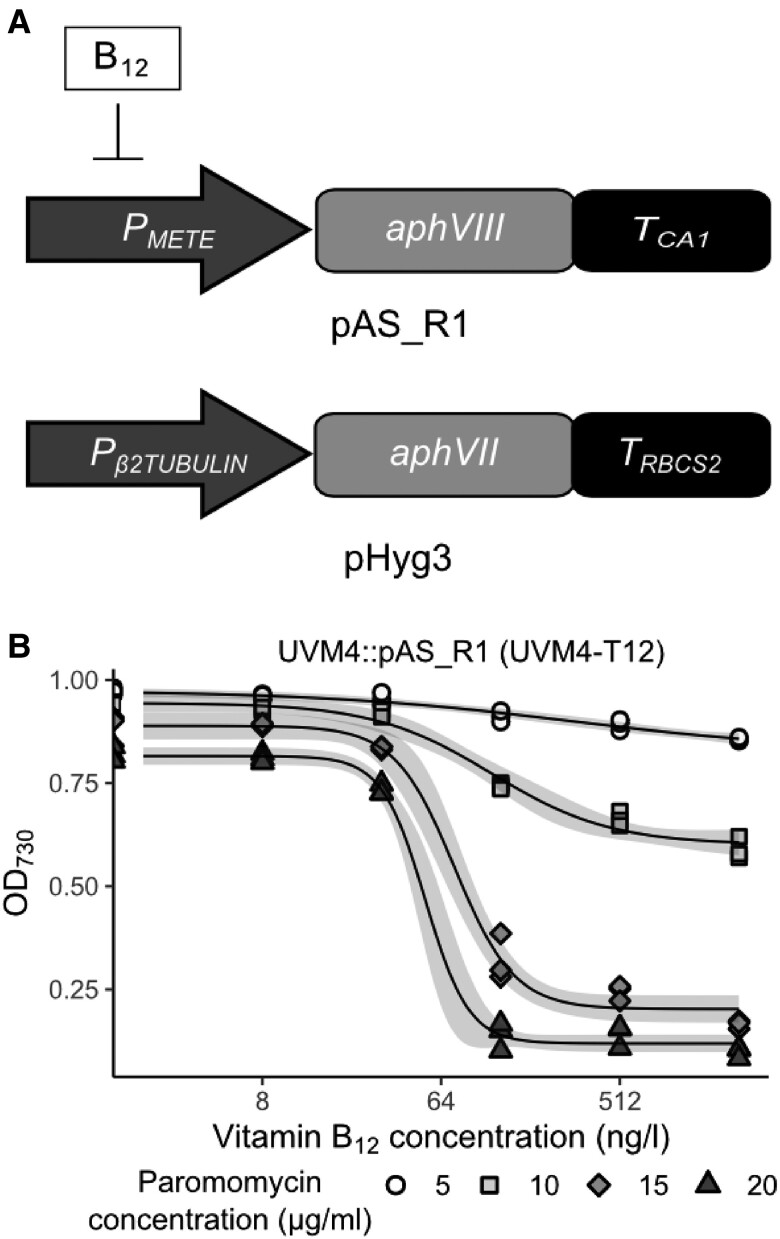
Generation and use of *C. reinhardtii* reporter strain UVM4-T12 for insertional mutagenesis. **A)** Schematic of the constructs used for insertional mutagenesis of *C. reinhardtii*. The pAS_R1 construct was designed to control expression of the paromomycin resistance gene (*aphVIII*) via B_12_ mediated repression of the *METE* promoter (*P_METE_*). The pHyg3 construct encoded a constitutively expressed hygromycin resistance gene (*aphVII*), to be used for insertional mutagenesis. **B)** Growth of *C. reinhardtii* B_12_ reporter strain UVM4-T12 bearing pAS_R1 plasmid, in response to vitamin B_12_ and paromomycin concentration in the media according to the algal dose–response assay. The predicted dose–response model is shown in black, with 95% confidence intervals in gray.

Bertrand et al. (2012) reported that there were no detectable *CBA1* homologues in algal lineages outside the Stramenopiles, so to investigate B_12_ uptake in *C. reinhardtii*, we decided to take an insertional mutagenesis approach to identify proteins involved in B_12_ uptake in *C. reinhardtii*. We took advantage of the fact that B_12_ represses expression of the *METE* gene at the transcriptional level via the promoter (*P_METE_*), and that reporter genes driven by this genetic element respond similarly (Helliwell et al. 2014), to develop a highly sensitive screen for lines no longer able to respond to B_12_. We hypothesized that, since *P_METE_* is likely to respond specifically to intracellular B_12_, *P_METE_* would not be repressed in strains unable to take up B_12_ from the media, so the reporter would be expressed and functional. If the reporter were an antibiotic resistance gene, this would allow identification of B_12_ uptake mutants in a more high-throughput manner than the B_12_-uptake assay. The background strain for insertional mutagenesis was made by transforming *C. reinhardtii* strain UVM4 (Neupert et al. 2009) with plasmid pAS_R1 containing a paromomycin resistance gene (*aphVIII*) under control of *P_METE_* (Fig. 2A, top construct; Supplemental Table S2). Lines of this strain were tested for their responsiveness to B_12_ and paromomycin. One line, UVM4-T12, showed the appropriate sensitivity with increasing repression of growth in paromomycin as B_12_ concentrations were increased, the effect being more marked at 15 to 20 µg·ml^−1^ paromomycin than at 5 to 10 µg·ml^−1^ (Fig. 2B). This line thus allowed for an easily quantifiable growth phenotype that was proportionally related to B_12_ concentration.

Insertional mutagenesis was carried out by transforming UVM4-T12 with a plasmid (pHyg3) containing a hygromycin resistance gene (*aphVII)* under the control of the constitutively expressed β2-tubulin promoter (Fig. 2A, bottom construct), generating a population of UVM4-T12::pHyg3 lines with the cassette randomly inserted into the nuclear genome. By plating the products of the transformation on solid TAP media supplemented with a range of paromomycin, hygromycin, and vitamin B_12_ concentrations (see Materials and Methods), seven colonies were obtained. This was from approximately 5,000 primary transformants, determined by plating the same volume on TAP plates with the antibiotics but without B_12_. These seven putative insertional mutant (IM) lines were then assessed for their ability to take up B_12_ using the B_12_ uptake assay. For UVM4, UVM4-T12, and insertional lines from the plate without B_12_ (labeled Control 1 to 3), similar amounts of B_12_ were recovered from the cellular and media fractions (Supplemental Fig. S1). This was also the case for six of the IM lines, suggesting that they could still take up B_12_ and were likely false positives of the initial screen. However, no B_12_ could be detected in the cellular fraction of UVM4-T12::pHyg3 #IM4 (hereafter referred to as IM4), indicating that this mutant line did not take up B_12_.

**Figure 3.**
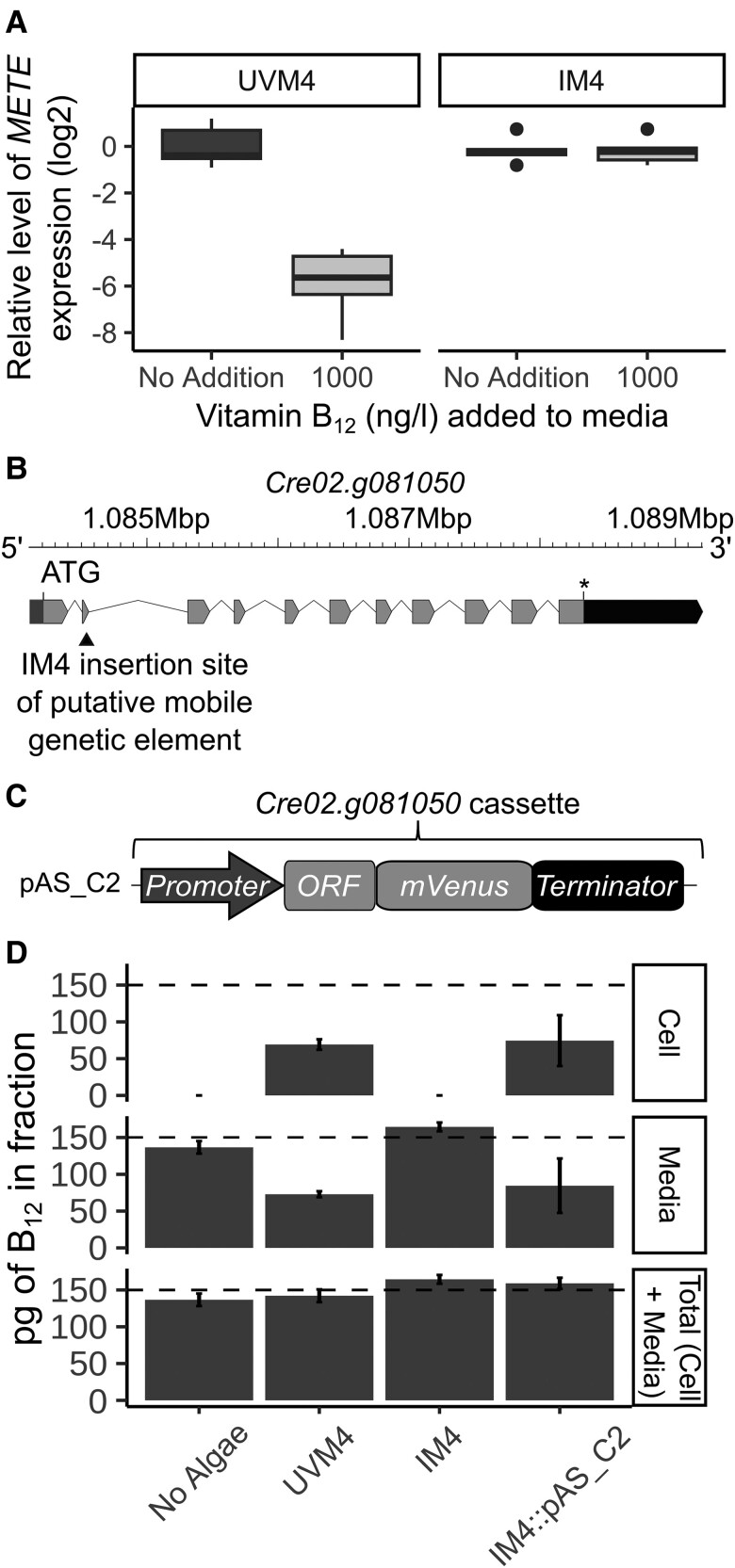
*C. reinhardtii* insertional mutant 4 (IM4) is defective in B_12_ response and uptake, and can be functionally complemented with *CrCBA1.***A)** Effect of vitamin B_12_ on *METE* gene expression in UVM4 and IM4, determined by RT-qPCR. UVM4 and IM4 were grown in TAP media with or without 1,000 ng·l^−1^ vitamin B_12_ for 4 d at 25 °C, 120 rpm and in continuous light (90 µE·m^−2^·s^−1^). Boxplots of the log_2_ transformed expression level of *METE* relative to that in the control (no B_12_) sample are shown, *n* = 6. The boxplots show the median (center line), 25th and 75th percentile hinges, and whiskers extending to the value no further than 1.5 times the interquartile range; values beyond this are plotted individually. Significant comparisons were identified using Tukey's test: UVM4 + 1,000 ng·l^−1^ vitamin B_12_ from UVM4 No Addition (*P* < 1e^−08^), IM4 No addition (*P* < 1e^−08^) and IM4 + 1,000 ng·l^−1^ vitamin B_12_ (*P* < 1e^−07^). **B)** Schematic of the Cre02.g081050 gene showing the position of the insertion site (indicated with a black triangle) determined by whole genome sequencing (Supplemental Fig. S4). **C)** Schematic of the pAS_C2 construct designed to express *CrCBA1* fused to the fluorescent reporter mVenus. *CrCBA1*-*mVenus* was under the control of the *CrCBA1* promoter and terminator. pAS_C2 also contained the spectinomycin resistance gene *aadA,* driven by the *PSAD* promoter and *PSAD* terminator. **D)** B_12_-uptake assay with UVM4, IM4 and IM4::pAS_C2 (*n* = 4 separate transformants with high mVenus expression). Dashed line indicates the amount of B_12_ added to the assay. Standard deviation error bars are shown. Statistical analysis was performed on the media fraction, and Tukey's test identified the following comparisons to be significantly different from one another: No Algae vs UVM4 (*P* < 1e^−05^); No Algae vs IM4 (*P* < 0.05); No Algae vs IM4::pAS_C2 (*P* < 1e^−03^); UVM4 vs 1.G2 (*P* < 1e^−09^); and 1.G2 vs 1.G2::pAS_C2 (*P* < 1e^−06^).

To obtain independent corroboration that IM4 was impaired in B_12_ uptake, cells of this mutagenized line were incubated with a fluorescently labeled B_12_ derivative, B_12_-BODIPY (Lawrence et al. 2018), and then imaged using confocal microscopy. *C. reinhardtii* cells were incubated in TAP medium without B_12_-BODIPY or with 1 µM B_12_-BODIPY for 1 h, washed with fresh media and subsequently imaged. There was no signal detected in the channel used for B_12_-BODIPY (589 nm excitation; 607 to 620 nm detection) in samples without B_12_-BODIPY added (Supplemental Fig. S2, top two rows), indicating that the imaging protocol was specific to this compound. When B_12_-BODIPY was added, UVM4-T12 showed the B_12_-BODIPY signal located within the algal cell (Supplemental Fig. S2, third row), indicating that this signal could be effectively detected by the imaging protocol and that B_12_-BODIPY was being transported into the cells. In contrast, there was no B_12_-BODIPY signal in IM4 cells, supporting the hypothesis that B_12_ uptake was impaired in this mutant (Supplemental Fig. S2, bottom row). In addition, the response of the *METE* gene to B_12_ in IM4 was assessed by RT-qPCR. UVM4 and IM4 cultures were grown in media with or without the addition of B_12_ for 4 d in continuous light, after which the cultures were harvested for RNA extraction and cDNA synthesis. As expected, *METE* was repressed in UVM4 in the presence of B_12_ compared to no supplementation (Fig. 3A), whereas IM4 showed similar *METE* expression in both conditions. This provided further support for disrupted B_12_ uptake in this line.

To identify the genomic location of the causal mutation in IM4, short-read whole genome sequencing was performed on DNA samples from UVM4, UVM4-T12, and IM4. The location of the pHyg3 cassette in IM4 was identified as described in the section Materials and Methods and found to have disrupted the *Cre12.g508644* locus (Supplemental Fig. S3A), an unannotated gene. To corroborate that disruption of the *Cre12.g508644* was responsible for the uptake-phenotype, two independent mutant lines of the gene (LMJ-119922 and LMJ-042227) were ordered from the Chlamydomonas library project (CLiP) collection (Li et al. 2016) and verified to be disrupted at this locus by PCR (Supplemental Fig. S3A). However, when these knockout lines were tested for the ability to take up B_12_ using the B_12_ uptake assay, they were both found to be able to do so to a similar extent as their parental strain, cw15 (Supplemental Fig. S3B). This suggested that *Cre12.g508644* did not encode a protein essential for B_12_ uptake.

We therefore examined the genome sequence data more closely to determine the genetic cause for the B_12_-uptake phenotype of IM4. We had identified putative homologues of human proteins involved in receptor-mediated endocytosis of B_12_, such as ABCD4, LMBD1 (Rutsch et al. 2009; Coelho et al. 2012), and MRP1 (Beedholm-Ebsen et al. 2010), in the *C. reinhardtii* genome by BLAST. However, given the widespread percentage of SNPs in the IM4 genome compared to UVM4, it was not possible to identify any candidate causal mutations with confidence. Instead, manual inspection of the DNA sequencing reads mapped to the reference strain revealed one locus, *Cre02.g081050,* annotated as flagella-associated protein 24 (FAP24), where there was a unique discontinuity in IM4, suggesting that there was an insertion at exon 2 in this gene (Fig. 3B; Supplemental Fig. S4A). The sequence was bordered by a genome duplication of 8 bp (shown in blue in Supplemental Fig. S4A) and exhibited imperfect inverted repeats at the terminal regions (TIRs), indicative of a transposable element. Reads could not be assembled across the discontinuity to obtain the complete sequence of the insertion, but using the left and right junction sequences as queries, three regions encoding two very similar genes were identified (Supplemental Fig. S4B).

Remarkably, when the *Cre02.g081050* protein was used as a query in a BLAST search, one of the hits recovered was the PtCBA1 protein (22.9% sequence identity), even though the reciprocal sequence search had not picked up the *C. reinhardtii* gene (Bertrand et al. 2012). Predicted 3D structures of PtCBA1 and the *C. reinhardtii* protein encoded by *Cre02.g081050* were obtained from the AlphaFold2 protein structure database and overlaid (Supplemental Fig. S5). The modeled proteins showed a high degree of structural similarity to one another (root mean squared deviation, RMSD = 1.688), particularly with respect to the arrangement of alpha helices and a lower cleft. Due to the sequence similarity and predicted structural similarity, these proteins appeared to be homologous to one another and Cre02.g081050 is hereafter referred to as CrCBA1.

To determine whether disruption of *CrCBA1* in IM4 was responsible for the impaired B_12_ uptake, we investigated whether it was possible to restore its ability to take up B_12_ by transforming IM4 with the WT *CrCBA1*. Construct pAS_C2 was designed with the *CrCBA1* promoter, *CrCBA1* open reading frame (ORF), and terminator and included a 3′ mVenus tag attached by a polyglycine linker (Fig. 3C). IM4 was transformed with pAS_C2, and resulting lines were tested for the ability to take up B_12_ using the B_12_ uptake assay. As observed previously, UVM4 was able to take up B_12_ whilst IM4 was unable to do so (Fig. 3D). The CBA1 complementation line IM4::pAS_C2 showed B_12_ in the cellular fraction at similar levels as in UVM4, thereby indicating that the mutant phenotype had been complemented.

### *CrCBA1* CLiP mutant is unable to take up B_12_ and is complemented by the WT *CrCBA1* gene

Given the many genetic changes in line IM4 compared to the parental UVM4-T12 strain caused by the mutagenesis, it was essential to have independent corroboration that mutation of *CrCBA1* caused the inability to take up B_12_. Accordingly, we obtained two further CLiP mutants (LMJ-135929 and LMJ-040682) with disruptions in intron 2 and introns 6/7, respectively of *CrCBA1* (Supplemental Fig. S6A) and assessed them for their ability to take up B_12_ (Supplemental Fig. S6B). No B_12_ was detected in cells of LMJ-040682, indicating complete inhibition of B_12_ uptake. Although LMJ-135929 cells accumulated some B_12_, this was less than half the amount of its parent strain cw15, suggesting partial impairment in uptake, similar to the phenotype of the monoallelic *PtCBA1* knockout line (Fig. 1C). However, heterozygosity cannot be the explanation for *C. reinhardtii,* which is haploid, and instead indicates that LMJ-135929 was likely to have just partial knockdown of the gene, probably because the insertion is in an intron.

**Figure 4.**
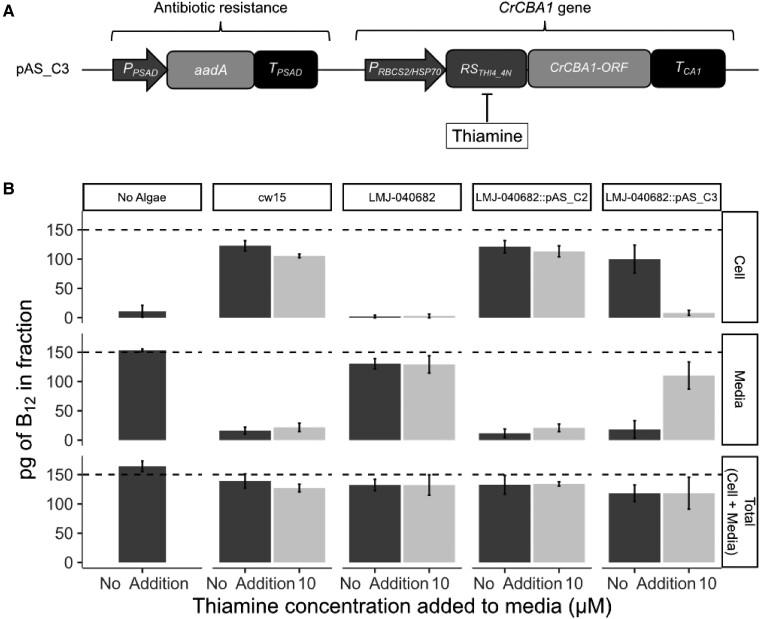
CLiP mutants in CrCBA1 are impaired in their ability to take up B_12_. **A)** Schematic of the pAS_C3 construct designed to express *CrCBA1* in a controllable manner using a thiamine repressible riboswitch (RS*_THI4_4N_*) to allow repression of *CrCBA1* through the addition of thiamine (Mehrshahi et al. 2020). **B)** B_12_-uptake assay with cw15, LMJ-040682 (mean of four independent transformants) and LMJ-040682::pAS_C2 and LMJ-040682::pAS_C3 (mean of three independent transformants). The growth conditions were modified compared to previous assays: lines were grown with or without 10 µM thiamine supplementation for 5 d in a 16/8 light/dark cycle, and 8 h after the dark to light transition the cultures were used for the algal B_12_-uptake assay. The dashed line indicates the amount of B_12_ added to the sample. Standard deviation error bars are shown. Statistical analysis was performed on the media fraction. Tukey's test identified the following algal strains to be significantly different from one another in media without thiamine (not reporting comparisons against the No Algae control condition): cw15 vs LMJ-040682 (*P* < 1e^−10^); LMJ-040682 vs LMJ-040682::pAS_C2 (*P* < 1e^−09^); and LMJ-040682 vs LMJ-040682::pAS_C3 (*P* < 1e^−09^). Additionally, Tukey's test found the following strain to show a significant difference due to thiamine addition: LMJ-040682::pAS_C3 (*P* < 1e^−07^).

Nonetheless, to provide further confirmation that mutations in *CrCBA1* were responsible for the observed impaired B_12_ uptake, we again tested whether the phenotype could be complemented with the WT *CrCBA1* gene using both plasmid pAS_C2 (Fig. 3B) and an additional construct pAS_C3 (Fig. 4A), in which expression of *CrCBA1* can be controlled by a thiamine pyrophosphate (TPP) repressible riboswitch, RS*_THI4_4N_* (Mehrshahi et al. 2020). In the absence of thiamine supplementation of the cultures, the riboswitch is not active and the gene containing it is transcribed and translated as normal; with thiamine addition, alternative splice sites are utilized, leading to inclusion of an upstream ORF containing a stop codon in the mRNA, preventing translation from the downstream start codon. LMJ-040682 was transformed with both pAS_C2 and pAS_C3, and representative transformant lines selected via antibiotic resistance were obtained. These, together with their parental strains were grown in the presence or absence of 10 µM thiamine for 5 d, and then used in the B_12_ uptake assay. Transformants of both LMJ-040682::pAS_C2 and LMJ-040682::pAS_C3 were found to take up B_12_ to a similar extent as their parental strain cw15 when grown in the absence of thiamine (Fig. 4B). However, when 10 µM thiamine was included in the culture medium, LMJ-040682::pAS_C3 showed virtually no B_12_ uptake. This riboswitch-mediated conditional complementation of the phenotype in LMJ-040682::pAS_C3 demonstrated conclusively that B_12_ uptake in *C. reinhardtii* is dependent on the presence of CrCBA1.

### CrCBA1 shows an association with membranes and is highly upregulated under B_12_-deprivation

To investigate the subcellular location of CrCBA1, we used several bioinformatic targeting prediction tools. CrCBA1 is annotated as a flagella-associated protein in the Phytozome v5.6 *C. reinhardtii* annotation. However, both DeepLoc (Almagro Armenteros et al. 2017) and SignalP (Almagro Armenteros et al. 2019), as well as AlphaFold2, indicated a hydrophobic sequence with the characteristics of a signal peptide at the N-terminus of CrCBA1 and predicted it would be targeted to the endoplasmic reticulum (ER). Additionally, it was predicted to contain a transmembrane helix at its C-terminus by InterPro (Mitchell et al. 2019) and AlphaFold2.

**Figure 5.**
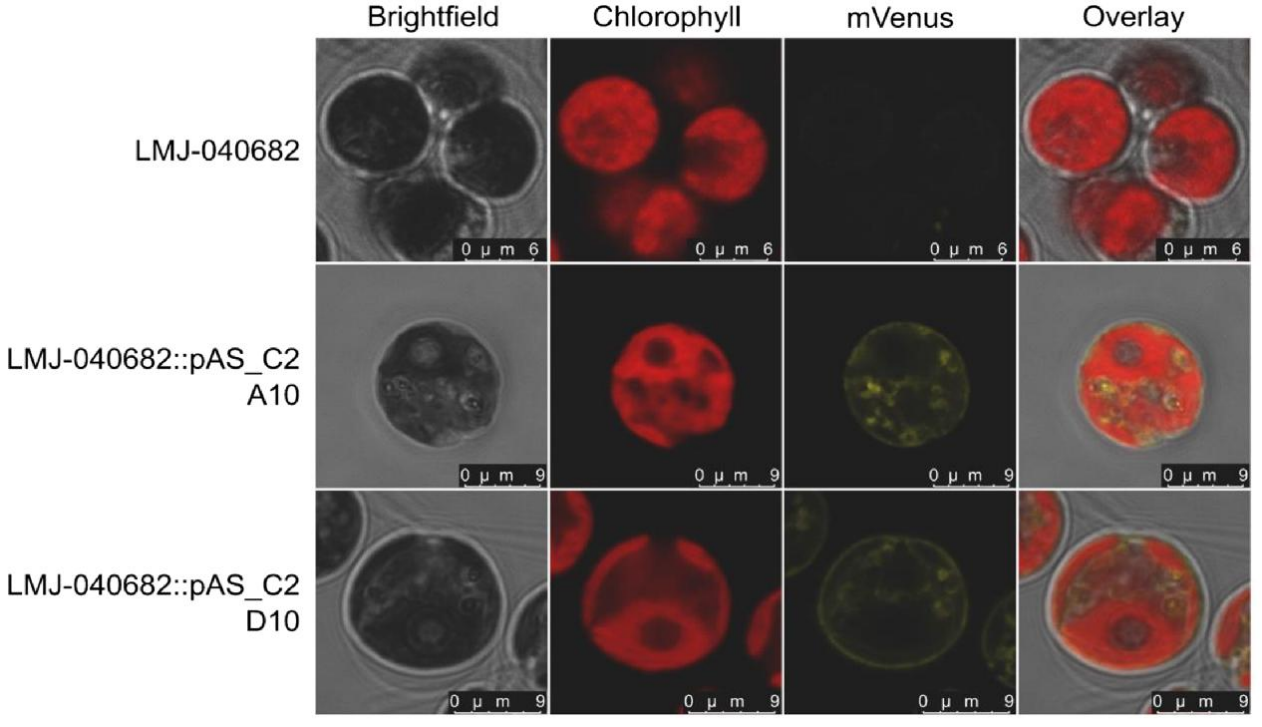
Confocal microscopy of complemented *C. reinhardtii CrCBA1* knockout lines showing an association between CrCBA1 and membranes. LMJ-040682 and LMJ-040682::pAS_C2 A10 and D10 lines were imaged according to the protocol outlined in the section Materials and Methods. Channels shown (left to right) are brightfield, chlorophyll, mVenus and an overlay. Microscope settings are described in the section Materials and Methods.

We next investigated the subcellular location of CrCBA1 in vivo by imaging two lines of LMJ-040682::pAS_C2, where the CBA1 is tagged with mVenus, with confocal microscopy. No mVenus was detected in the parental LMJ-040682 cells, whereas a clear fluorescent signal was observed in LMJ-040682::pAS_C2 #A10 and LMJ-040682::pAS_C2 #D10 (Fig. 5). In these complemented lines, the mVenus signal was absent from the chloroplast, nucleus, and flagella, but instead could be seen within the cell localizing both to the plasma membrane and to regions that may be endomembranes such as the ER. This is consistent with findings from *P. tricornutum* showing a similar distribution (Bertrand et al. 2012). Together these data indicate that CBA1 is likely to be associated with membranes, and therefore, may have a conserved role in the B_12_ uptake process.

**Figure 6.**
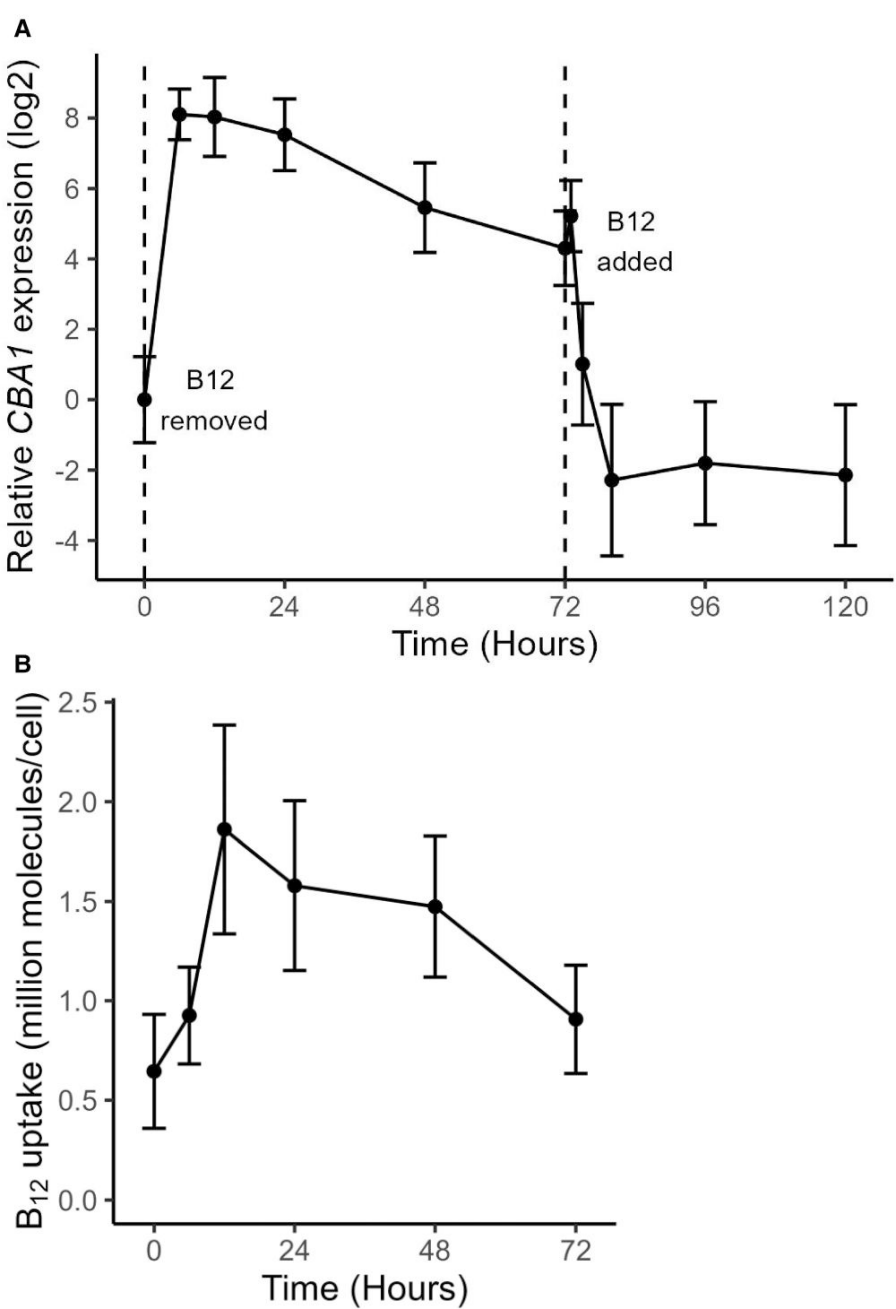
*CBA1* expression and B_12_ uptake capacity in a B_12_-dependent mutant of *C. reinhardtii* (metE7) during B_12_ starvation and add-back. **A)** Log_2_ transformed expression level of CBA1 measured by RT-qPCR and presented relative to levels in control conditions (B_12_ replete). Vertical dashed lines denote when B_12_ was removed and added. **B)** B_12_ uptake capacity of starved metE7 cells (expressed as 10^6^ molecules of B_12_ per cell over 1 h) at the same six time points during B_12_ starvation; it was not possible to perform the uptake assay on cells to which B_12_ had already been added. Cell density measurements were performed by counting plated cells in dilution series, and so included nonviable cells. For CBA1 expression and B_12_ uptake, three and six biological replicates were used, respectively, with points representing means, and error bars representing standard deviations.

Further evidence for the role of CBA1 in B_12_ uptake was obtained by taking advantage of a B_12_-dependent mutant of *C. reinhardtii*, metE7 (Helliwell et al. 2015; Bunbury et al. 2020). We tested the effect of B_12_-deprivation over time on the expression of the *CrCBA1* gene by RT-qPCR in the mutant and determined the rate of B_12_ uptake over a similar period. Within 6 h of B_12_ removal, there was a ∼250-fold induction of the *CrCBA1* transcript, followed by a slow decline over the next 60 h (Fig. 6A). After resupply of B_12_ there was then a rapid ∼100-fold decline within 8 h. The B_12_ uptake capacity of metE7 followed a similar profile, increasing 3-fold over the first 12 h of B_12_ depletion, from ∼6.5 × 10^5^ molecules B_12_/cell/h to 1.86 × 10^6^ molecules B_12_/cell/h (Fig. 6B), then declining slowly. This induction profile is characteristic of a nutrient-starvation response shown by many transporters, including in *C. reinhardtii* those for Fe (Allen et al. 2007), and for *CBA1* in the B_12_-dependent diatom, *T. pseudonana* (Bertrand et al. 2012).

### Widespread distribution of CBA1 in algae

Having shown the importance of *PtCBA1* and *CrCBA1* for B_12_ uptake in their respective species, we re-examined how prevalent CBA1-like proteins are in nature. Searches with BLASTP using *PtCBA1* were reported to result in no significant hits in species outside the Stramenopiles (Bertrand et al. 2012). Instead, we created a hidden Markov model (HMM), using the *C. reinhardtii* CBA1 amino acid sequence and CBA1 sequences from *P. tricornutum*, *T. pseudonana*, *Fragilariopsis cylindrus*, *Aureococcus anophagefferens*, and *Ectocarpus siliculosus* (Bertrand et al. 2012), to identify more accurately CBA1-like proteins in other organisms. The EukProt database of curated eukaryotic genomes (Richter et al. 2022) includes representatives from the Archaeplastida (designated by EukProt as Chloroplastida), which encompass green algae, red algae, glaucophytes and all land plants, as well as phyla that include algae with complex plastids, namely Stramenopiles (which include diatoms), Alveolata (including dinoflagellates), Rhizaria, and Haptophyta, and the animals (both Metazoa and the Choanoflagellates, unicellular and colonial flagellated organisms considered to be the closest living relatives of the animals (King et al. 2008)), the fungi and Amoebozoa. This database was queried with the CBA1 HMM model, using a cutoff *e*-value of 1*e–*20, and 277 hits were obtained (Supplemental Fig. S7; Supplemental Table S4). No candidates were found in the Metazoa, but CBA1 homologues were identified in all other phyla, including all photosynthetic groups, fungi and amoebozoa and in choanoflagellates.

**Figure 7.**
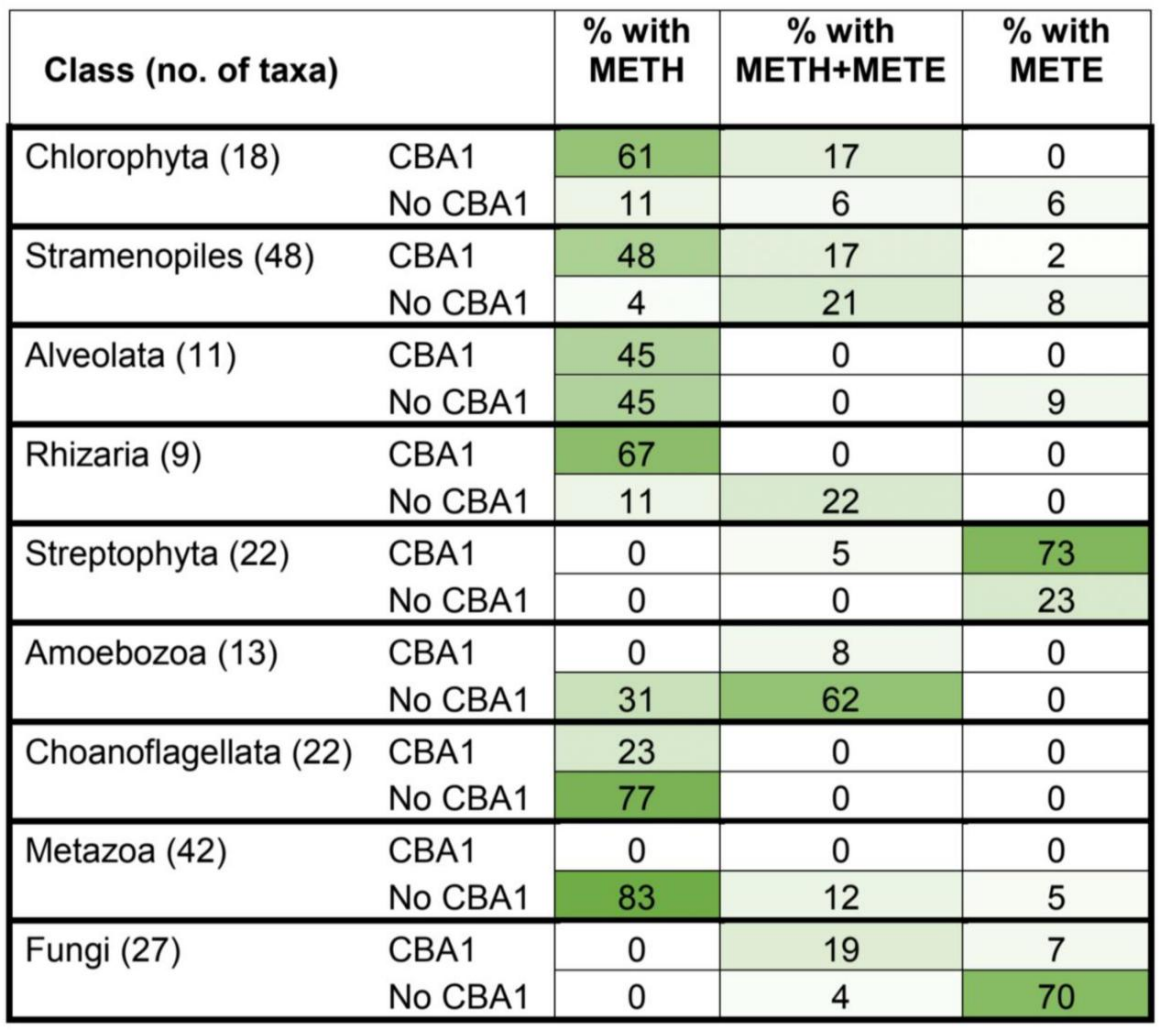
Distribution of CBA1 and methionine synthase sequences across Eukaryotic groups. The EukProt database (Richter et al. 2022) was searched for METE, METH, and CBA1 queries, as described in the section Materials and Methods. Organisms were only considered if they contained at least one valid methionine synthase hit (METE or METH) and their genomes were >70% complete, as measured by BUSCO (Manni et al. 2021). Eukaryotic classes were filtered for those with greater than five genomes and the numbers of taxa for each class are indicated in brackets. The different combinations of CBA1, METE, and METH were calculated for each species (Supplemental Table S4) and summarized as a percentage of the total number of taxa in each class, with gradual shading to show the variation in distribution between the different classes.

Given that vascular plants have no B_12_-dependent enzymes, the presence of a putative B_12_-binding protein in several angiosperms, both monocot and dicot, and the gymnosperm *Ginkgo biloba*, was somewhat surprising. To address this conundrum, we investigated to what extent CBA1 was associated with vitamin B_12_ dependence by determining the distribution of the different isoforms of METH and METE. Using the same HMM approach as before, the protein sequences were searched against the EukProt database and the combination of presence and absence of CBA1, METH, and METE across eukaryotic species groups was compiled (Fig. 7; Supplemental Table S5). What is immediately apparent is that the combination of the three proteins is quite different in the various lineages. In the major algal groups, the Chlorophyta and the SAR clade (Stramenopiles, Alveolata, and Rhizaria), METH sequences were found in the majority of genomes analyzed and their presence was associated with CBA1. In the genomes of the Chlorophyta and the SAR clade that encoded METE only (seven taxa in total), CBA1 was absent in all but one, the diatom *Thalassionema nitzschiodes*. Equal numbers of Alveolata species encoded METH and CBA1, or METH only; interestingly, the latter were all nonphotosynthetic lineages. Grouping the data from these four algal groups, a Chi Square test was significant for CBA1 and METH being more often both present or both absent (*X2* (1, *N* = 86) = 9.2, *P* = 0.00240). The association could be due to linkage, although in neither *C. reinhardtii* nor *P. tricornutum* are the two genes on the same chromosome, making this unlikely. Alternatively, there is a fitness advantage in both genes being acquired or lost together.

Most fungal taxa lacked both METH and CBA1, but we found examples of six species that were predicted to be B_12_ users (METH present) and five of these were also predicted to contain CBA1-like sequences: *Allomyces macrogynus*, *Spizellomyces punctatus*, *Rhizophagus irregularis*, *Rhizopus delemar*, and *Phycomyces blakesleeanus*. CBA1-like sequences were identified in the Opisthokonta and Amoebozoa, although were less prevalent, with ∼23% of choanoflagellates and 8% of amoeboid species being like algae in having both METH and CBA1. CBA1 was entirely absent from the Metazoa. In contrast, in the Streptophyta, which include multicellular green algae and all land plants, the majority lack METH, but almost 80% of species were found to contain CBA1-like sequences. This implies that Streptophyta CBA1 sequences may have gained a different function, which would be consistent with the lack of B_12_-dependent metabolism in these organisms. In summary, these data suggest that CBA1 is associated with vitamin B_12_ use to different degrees in different eukaryotic groups, with there being a greater association in obligate and facultative B_12_ users than in those organisms that do not utilize B_12_.

**Figure 8.**
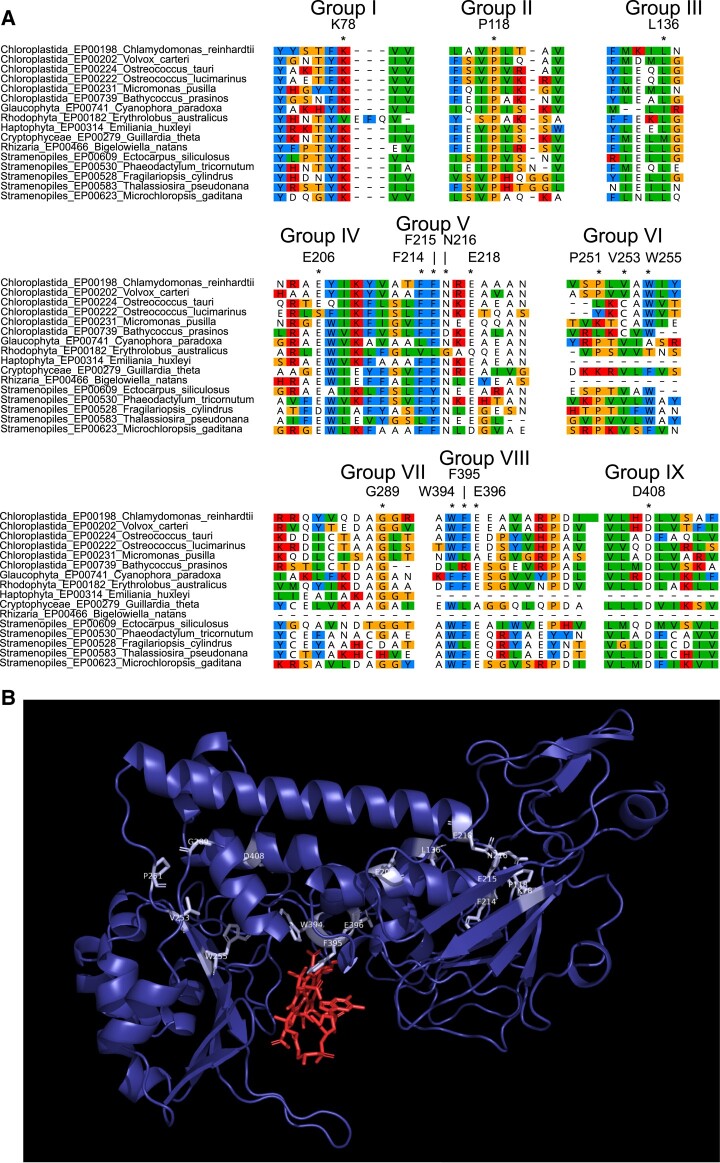
Identification and predicted structural location of CrCBA1 conserved residues. **A)** Sequences with similarity to CBA1 were identified from the EukProt database (Richter et al. 2022) using a manually generated CBA1 HMM, as described in the section Materials and Methods. A selection of 16 taxa from several eukaryotic supergroups were chosen and conserved regions from the protein are presented. Specific residues indicated by * are: K78, P118, L136, E206, F214, F215, N216, E218, P251, V253, W255, G289, W394, F395, E396, and D408. Protein sequences are colored according to the Clustal color-scheme using Geneious Prime 2021.1.1 (www.geneious.com). For each highly conserved region, the corresponding position, and amino acid from the CrCBA1 sequence (Cre02.g081050) is indicated. **B)** The predicted 3D structure of CrCBA1 (residues 21-490) was obtained from the AlphaFold Protein Structure Database (entry: A0A2K3E0J7). Highly conserved regions of CrCBA1 are indicated in light blue and labeled. CrCBA1 was aligned to the crystal structure of *E. coli* BtuF in complex with B12 (pdb: 1n2z). This enabled the relative position of B12 (shown in red) to be superimposed onto CrCBA1.

The many putative CBA1 homologues in algal lineages and their strong association with B_12_ uptake provided an opportunity to identify conserved, and thus likely functionally important, residues. Accordingly, a multiple sequence alignment of proteins matching the CBA1 HMM query was generated (Supplemental Fig. S7). Highlighted in green in the similarity matrix at the top are nine conserved regions with several almost completely conserved residues; these are shown in more detail in Fig. 8A for selected taxa representing different algal groups. Further insight came from inspection of the model of the 3D structure of CrCBA1 generated by the AlphaFold2 protein structure database. The analysis showed that regions of CrCBA1 showed similarity to bacterial periplasmic binding proteins, including the B_12_-binding protein BtuF. A structure is available of *E. coli* BtuF in complex with B_12_ (Borths et al. 2002), so we compared this to the modeled CrCBA1 structure. Although there is little sequence similarity, alignment of the two structures resulted in an RMSD of 3.362 and enabled the relative position of B_12_ to be placed in the lower cleft of CrCBA1, shown in red in Fig. 8B. Mapping of the highly conserved residues onto this structure found that four (W255, W394, F395, and E396) were in a cluster around the relative position of B_12_. Another cluster of highly conserved residues were located at the end of the upper alpha helix (K78, P118, L136, F214, F215, N216, and E218). Both clusters represent promising mutational targets to investigate CrCBA1 function.

## Discussion

In this study, we have shown experimentally that a conserved protein, CBA1, is required for the uptake of the micronutrient B_12_ in two taxonomically distant algae, the diatom *P. tricornutum* (Fig. 1) and the chlorophyte *C. reinhardtii* (Figs. 3 and 4). Strains with knockouts of the gene were unable to take up B_12,_ demonstrating that there is no functional redundancy of this protein in either organism. As well as providing evidence that CBA1 is present outside the Stramenopiles, we found widespread occurrence of CBA1 homologues with considerable sequence conservation across eukaryotic lineages (Fig. 7; Supplemental S7). The strong association of CBA1 with the B_12_-dependent methionine synthase, METH, in algal lineages provides evidence that CBA1 is a key component of the B_12_ uptake process in evolutionarily distinct microalgae, and the structural similarities between CBA1 and BtuF (Fig. 8B) suggest it may operate as a B_12_-binding protein. The highly conserved residues identified in the algal homologues (Fig. 8A) offer the means to determine which are functionally important, facilitated by the uptake assay we established.

Nonetheless, the mechanistic role of CBA1 in the process of B_12_ acquisition in algae is not yet clear. Previous physiological studies of B_12_ uptake by microalgae, such as the haptophyte *Diacronema lutheri* (Droop 1968), indicated a biphasic process: firstly, rapid irreversible adsorption of B_12_ to the cell exterior, followed by a slower second step of B_12_ uptake into the cell, consistent with endocytosis. CBA1 is unlikely to be associated with the binding of B_12_ in the cell wall, however. This is because the *C. reinhardtii* strains used in this study, UVM4 and CW15, were cell wall deficient, and therefore likely also deficient in cell wall proteins that bind B_12_; the lack of a B_12_-BODIPY signal from the cell surface in IM4 (Supplemental Fig. S2) supports this hypothesis. Further use of this fluorescent probe offers the possibility to monitor the localization of B_12_-BODIPIY over time to gain insights into the stages of B_12_ uptake, as has been done in other organisms (Lawrence et al. 2018). In addition, confocal microscopy of CBA1-mVenus fusion protein in *C. reinhardtii* (Fig. 5) showed an apparent association of CrCBA1 with the plasma membrane and endomembranes, which is similar to that for ER-localized proteins (Mackinder et al. 2017). Moreover, in a proteomics study of lipid droplets (which form by budding from the ER) CBA1 was in the top 20 most abundant proteins (Goold et al. 2016). Bertrand et al. (2012) found that PtCBA1 had a signal peptide and fluorescently tagged PtCBA1 was also targeted to the ER. Nonetheless, based on its predicted 3D structure and the fact that it has at most one transmembrane helix, CBA1 does not appear to be a transporter itself. Instead, given its structural similarity to BtuF, a distinct possibility is that CBA1 is the soluble component of an ABC transporter, either at the plasma membrane or an internal membrane, and likely will interact with one or more other proteins to allow B_12_ uptake to occur, at least some of them being those involved in receptor-mediated endocytosis, as is the case for B_12_ acquisition in humans (Rutsch et al. 2009; Beedholm-Ebsen et al. 2010; Coelho et al. 2012). In this context, there are known similarities between endocytosis in *C. reinhardtii* and humans (Denning and Fulton 1989; Bykov et al. 2017), and several putative homologues have been identified by sequence similarity in the alga. Testing the B_12_-uptake capacity of mutants of these proteins would be one approach to investigate whether their roles are also conserved.

In contrast to the situation in algae, the Streptophyta live in a B_12_-free world, neither synthesizing nor utilizing this cofactor. This is exemplified by the fact that in our analysis only one species, the charophyte alga *Cylindrocystis brebissonii*, encoded METH. Despite this, more than three-quarters of this group encode a CBA1 homologue (Fig. 7;Supplemental Fig. S7). Since the majority of the conserved residues (Fig. 8A) are also found in putative CBA1 sequences in the angiosperms such as *Arabidopsis*, including those around the potential binding pocket, it is possible that the streptophyte protein has acquired a new function that still binds a tetrapyrrole molecule. Intriguingly, the reverse is observed in the Metazoa, where METH is almost universal, but CBA1 is entirely absent. However, some Choanoflagellates and some species of fungi do appear to encode both METH and CBA1, suggesting that they utilize B_12_, a trait recognized to occur in fungi only recently (Orłowska et al. 2021). It will be of interest, therefore, to test whether CBA1 is involved in B_12_ uptake in these organisms, for example by gene knockout studies.

The importance of B_12_ availability for phytoplankton productivity has been demonstrated across several marine ecosystems by amendment experiments (e.g. Bertrand et al. 2011; Koch et al. 2012; Joglar et al. 2021), where addition of B_12_ led to algal blooms and affected the composition and stability of microbial communities. The mode of acquisition of this micronutrient is thus likely to be highly conserved and subject to substantial ecological and evolutionary selection pressure to be retained. Moreover, the role of B_12_ at the cellular level may well provide a direct connection between environmental conditions and the epigenetic status of the genome: methionine synthase is the key enzyme in C1 metabolism, linking the folate and methylation cycles and thus responsible for maintaining levels of S-adenosylmethionine, the universal methyl donor (Hanson and Roje 2001; Mentch and Locasale 2016). In this context, it is noteworthy that the knockout of *CBA1* in the IM4 line was the result of insertion of a class II transposable element into the gene. This mobilization is likely to reflect epigenetic alterations of the autonomous element, presumably as a result of cellular stress from the antibiotic selection, or from the transformation procedure, or both. Recent classification of the transposons in *C. reinhardtii* indicate that the transposon inserted into *CBA1* in IM4 is a member of the KDZ superfamily of class II TIR elements named Kyakuja-3_cRei (Craig et al. 2021). If the phenomenon of inactivation of a gene that is deleterious (in this case allowing B_12_ to be taken up and repress the antibiotic resistance gene) via transposition is a general response in *C. reinhardtii*, repeating the screen for CBA1 mutants might allow observation of further transposition events, and enable characterization of this group of elements at the functional level. Moreover, it could be adopted as a more general methodology to identify candidate genes involved in other physiological processes, by tying their expected effects to deleterious outcomes through synthetic biology constructs and screening surviving mutants by sequencing.

## Materials and methods

### Organisms and growth conditions

Strains, media, and growth conditions used in this study are listed in Supplemental Table S1. If required, antibiotics, vitamin B_12_ (cyanocobalamin), and thiamine were added to the medium at concentrations indicated. Algal culture density was measured using a Z2 particle count analyzer (Beckman Coulter Ltd.) and optical density (OD) at 730 nm was measured using a FluoStar OPTIMA (BMG labtech) plate reader or a CLARIOstar plate reader (BMG labtech). Bacterial growth was recorded by measuring OD_595_.

### Algal B_12_-uptake assay

Algal cultures were grown to stationary phase and cyanocobalamin salt (Sigma-Aldrich, Dorset, UK) was added (*P. tricornutum*: 600 pg; *C. reinhardtii*: 150 pg) to 5 × 10^6^ cells in a final volume of 1 ml in *f*/2 or TAP medium, respectively. The samples were incubated at 25 °C under continuous light with shaking for 1 h and inverted every 30 min to aid mixing. Samples were centrifuged and the supernatant (media fraction) transferred into a fresh microcentrifuge tube. The cell pellet was resuspended in 1 ml water. Both samples were boiled for 10 to 20 min to release any cellular or bound B_12_ into solution, and then centrifuged to pellet debris. The supernatant was used in the *S. typhimurium* B_12_ bioassay as described in Bunbury et al. (2020). The amount of B_12_ in the sample was calculated by comparison to a standard curve of known B_12_ concentrations fitted to a four-parameter logistic equation *f*(*x*) = *c* + (*d − c*)(1 + exp(*b*(log(*x*) *−* log(*e*)))) (Ritz et al. 2015). This standard curve was regenerated with every bioassay experiment.

### Generating *P. tricornutum* CBA1 knockout lines using CRISPR-Cas9

CRISPR-Cas9 genome editing applied the single guide RNA (sgRNA) design strategy described in Hopes et al. (2017). Details are provided in the Supplemental methods. *P. tricornutum CCAP 1055/1* cells were co-transformed with linearized plasmids pMLP2117 and pMLP2127 (Supplemental Table S2) using a NEPA21 Type II electroporator (Nepa Gene) as previously described (Yu et al. 2021). After plating on 1% agar selection plates containing 75 mg·l^−1^ zeocin and incubation for 2 to 3 wk, zeocin resistant colonies were picked into 96 well plates containing 200 µl of *f*/2 media with 75 mg·l^−1^ zeocin. After seven days strains were subcultured into fresh media either containing 75 mg·l^−1^ zeocin or 300 mg·l^−1^ nourseothricin, and genotyped with a three-primer PCR using PHIRE polymerase (Thermo Fisher Scientific) with primers gCBA1.fwd, gCBA1.rv and NAT.rv (Supplemental Table S3). Five promising colonies resistant to nourseothricin and with genotypes showing homologous recombination or indels were re-streaked on 75 mg·l^−1^ zeocin *f*/2 plates to obtain secondary monoclonal colonies. Twelve secondary colonies were picked for each primary colony after 2 to 3 wk and again genotyped with a three-primer PCR. Promising colonies were genotyped in further detail with primer pairs gCBA1.fwd/gCBA1.rv, gCBA1.fwd/NAT.rv and gCBA1in.fwd/gCBA1in.rv (Supplemental Table S3).

### Construct assembly and *C. reinhardtii* transformation

Constructs were generated using Golden Gate cloning, using parts from the *Chlamydomonas* MoClo toolkit (Crozet et al. 2018) and some that were created in this work. All parts relating to *Cre02.g081050* were domesticated from UVM4 genomic DNA, with BpiI and BsaI sites removed from the promoter, ORF and terminator by PCR-based mutagenesis using primers listed in Supplemental Table S3. A list of plasmids used in this study is shown in Supplemental Table S2. Transformation of *C. reinhardtii* cultures with linearized DNA was carried out by electroporation essentially as described by Mehrshahi et al. (2020) before plating on TAP-agar plates with the appropriate antibiotics.

Insertional mutagenesis was performed as above, however, cultures were grown to a density of approximately 1 × 10^7^ cells/ml and were incubated with 500 ng transgene cassette. After allowing the cells to recover overnight in TAP plus 60 mM sucrose at 25 °C in low light (less than 10 μmol·photon·m^−2^·s^−1^ at 100 rpm), between 200 and 250 μl of transformants were plated on solid TAP media (square 12 × 12 cm^2^ petri dishes) containing ranges of 15 to 20 μg/ml hygromycin, 20 to 50 μg/ml paromomycin, and 48 to 1,024 ng/l vitamin B_12_, and the plates were incubated in standing incubators.

### Confocal laser scanning microscopy

*C. reinhardtii* transformants carrying the pAS_C2 construct were imaged in a confocal laser scanning microscope (TCS SP8, Leica Microsystems, Germany) with an HC PL APO CS2 40×/1.30 aperture oil-immersion lens. Images were taken using the sequential mode provided by the Leica LAS software, with the channel used for mVenus and brightfield detection being taken first and the channel used for chlorophyll detection taken second. The first image was acquired with excitation from a white light source at 486 nm at 7% power and emissions were detected between 520 and 567 nm; mVenus settings included 100% gain, gating between 0 and 8.24 ns and a reference line at 486 nm. Brightfield imaging used 610% gain and a 0% offset. Frames were captured with a line average of 4 and a frame accumulation of 2. The second image was acquired with excitation from a white light source at 514 nm at 2% power and emissions were detected between 687 and 724 nm with 50% gain. Frames were captured with a line average of 4 and a frame accumulation of 1. The overlay images were produced automatically by the Leica LAS software. Inkscape was used to increase the lightness and decrease the contrast of all the images in the same manner.

### Reverse transcription quantitative PCR

Quantification of steady-state levels of transcripts was carried out according to Bunbury et al. (2020), using random hexamer primers for cDNA synthesis. The RT-qPCR data was analyzed using the ΔΔCT method with an assumed amplification efficiency of 2. -ΔCT values relative to a control condition were plotted in the resulting figures.

### Whole genome sequencing

Genomic DNA was extracted from *C. reinhardtii* cells by phenol–chloroform extraction and sequenced using the NovaSeq sequencing platform by Novogene (Cambridge, UK) to produce 150 bp paired-end reads. This involved RNase treatment and library preparation with the NEBNext Ultra II DNA Library Prep Kit (PCR-free), which generated 350 bp inserts. The raw sequencing data for this study have been deposited in the European Nucleotide Archive (ENA) at EMBL-EBI under accession number PRJEB58730 (https://www.ebi.ac.uk/ena/browser/view/PRJEB58730). Novogene performed all quality filtering, summary statistics, and bioinformatic analysis. The location of the Hyg3 cassette was determined by identifying loci that comprised reads from IM4 that mapped between genomic DNA and pHyg3, and cross-referencing these loci against the parental strains. The TE identification was carried out similarly, full details are provided in the Supplemental Methods.

### Bioinformatics pipeline

The EukProt database was assessed for the presence of METE, METH, and CBA1 (Richter et al. 2022). The query used for CBA1 was a HMM generated from the protein fasta sequences: Phatr3_J48322, Thaps3 11697, Fracy1 241429, Fracy1 246327, Auran1 63075, *E. siliculosus* D8LMT1, and Cre02.g081050.t1.2 by first aligning using MAFFT (Katoh and Standley 2013) version 7.470 with the auto option, and then building a HMM using hmmbuild (hmmer 3.2.1). Additionally, queries of protein fasta (Cre06.g250902, Cre03.g180750), curated protein families (PFAM) (PF02310, PF02965, PF00809, PF02574, PF01717, PF08267), and KEGG orthologue (KO) (K00548, K00549) were searched against EukProt to identify sequences with similarity to METE and METH. The queries were searched against EukProt using hmmsearch (HMMER 3.1b2). The default bitscore thresholds were used for KO and PFAM queries. The threshold used for CBA1 HMM, and the CrMETE and CrMETH protein fasta sequences, was a full-length *e*-value of 1e−20. For each protein, all individual queries were required to be significant to classify the protein as present. The best hit in each species was identified by taking the protein with the greatest geometric mean of full length bitscores for the queries. The dataset was joined with taxonomic information from EukProt and completeness information calculated using BUSCO version 4.1.4 and eukaryote_odb10 (Manni et al. 2021).

### Accession numbers

The data underlying this article are available in the GenBank/EMBL data libraries under accession number PRJEB58730 (https://www.ebi.ac.uk/ena/browser/view/PRJEB58730) and in its online supplementary material.

## Supplementary Material

kiad564_Supplementary_Data

## Supplemental data

The following materials are available in the online version of this article.

**Supplemental Figure S1.** Characterization of B_12_ uptake in *C. reinhardtii* insertional mutant lines.

**Supplemental Figure S2.** Visualization of B_12_-BODIPY uptake in *C. reinhardtii* using confocal microscopy.

**Supplemental Figure S3.** *C. reinhardtii* knockout lines of *Cre12.g508644* are able to take up B_12_.

**Supplemental Figure S4.** Structure and sequence of a second insertion in the IM4 strain.

**Supplemental Figure S5.** The predicted structures of CrCBA1 and PtCBA1 show a high degree of structural similarity.

**Supplemental Figure S6.** Independent mutant lines of *CrCBA1* show defective B_12_ uptake.

**Supplemental Figure S7.** Sequences with similarity to CBA1 are found throughout Eukaryota.

**Supplemental Table S1.** Table of strains used in this work.

**Supplemental Table S2.** Plasmids used in this study.

**Supplemental Table S3.** Oligonucleotides used in this study.

**Supplemental Table S4.** Protein sequences with similarity to CBA1 used for tree building in Supplemental Fig. S7.

**Supplemental Table S5.** Summary of CBA1, METE, and METH presence/absence found in organisms within Supergroups with greater than five organisms with a completeness greater than 70% and at least one of: METE or METH.
