# Supplementary material for: Conserved cobalamin acquisition protein 1 is essential for vitamin B12 uptake in both Chlamydomonas and Phaeodactylum
Source: Plant Physiol. 2023 Oct 21;194(2):698–714. doi: 10.1093/plphys/kiad564 (PMC10828217; doi:10.1093/plphys/kiad564)
Supplement: kiad564_Supplementary_Data [file kiad564_supplementary_data.zip › Sayer et al Supplemental material revised 15Oct23.pdf]

Supplemental Material

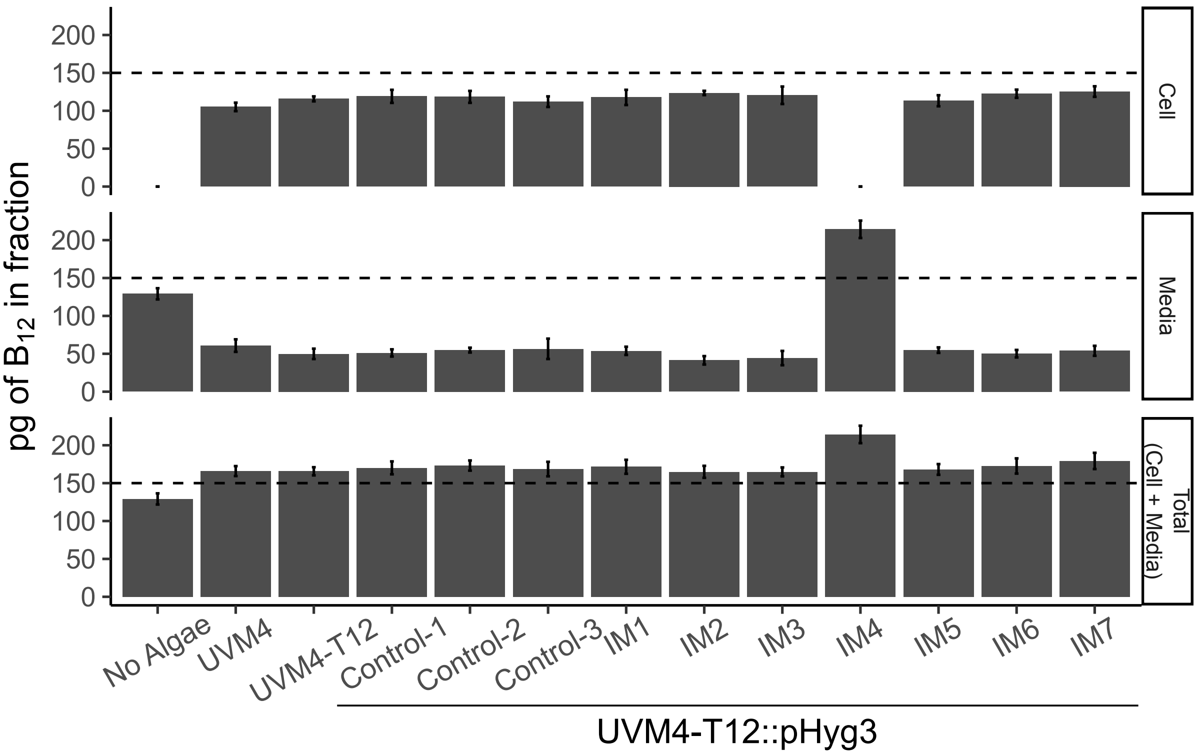

Figure S1. Characterization of B_{12} uptake in *C. reinhardtii* insertional mutant lines. To determine whether any of the 7 insertional mutant lines (IM) isolated from mutagenesis of UVM4::T12 showed impaired B_{12} uptake, the B_{12}-uptake assay was performed as described in the materials and methods. Control-1, Control-2 and Control-3 lines were picked from solid media with paromomycin and hygromycin but without vitamin B_{12}, whereas IM1-IM7 lines were picked from solid media containing paromomycin, hygromycin and vitamin B_{12}. The total was inferred by the addition of the cell and media fractions. The dashed line indicates the amount of B_{12} added in the uptake assay. Standard deviation error bars are shown, n=4. Statistical analysis was performed on the media fraction, and Tukey's test identified the following comparisons to be significantly different from one another (only reporting IM strains different from UVM4, UVM4-T12 or Control-[1,2,3] strains): IM4 vs UVM4 ($p<1e^{-12}$), UVM4-T12 ($p<1e^{-12}$), Control-1 ($p<1e^{-12}$), Control-2 ($p<1e^{-12}$), Control-3 ($p<1e^{-12}$); and IM2 vs UVM4 ($p<0.05$).

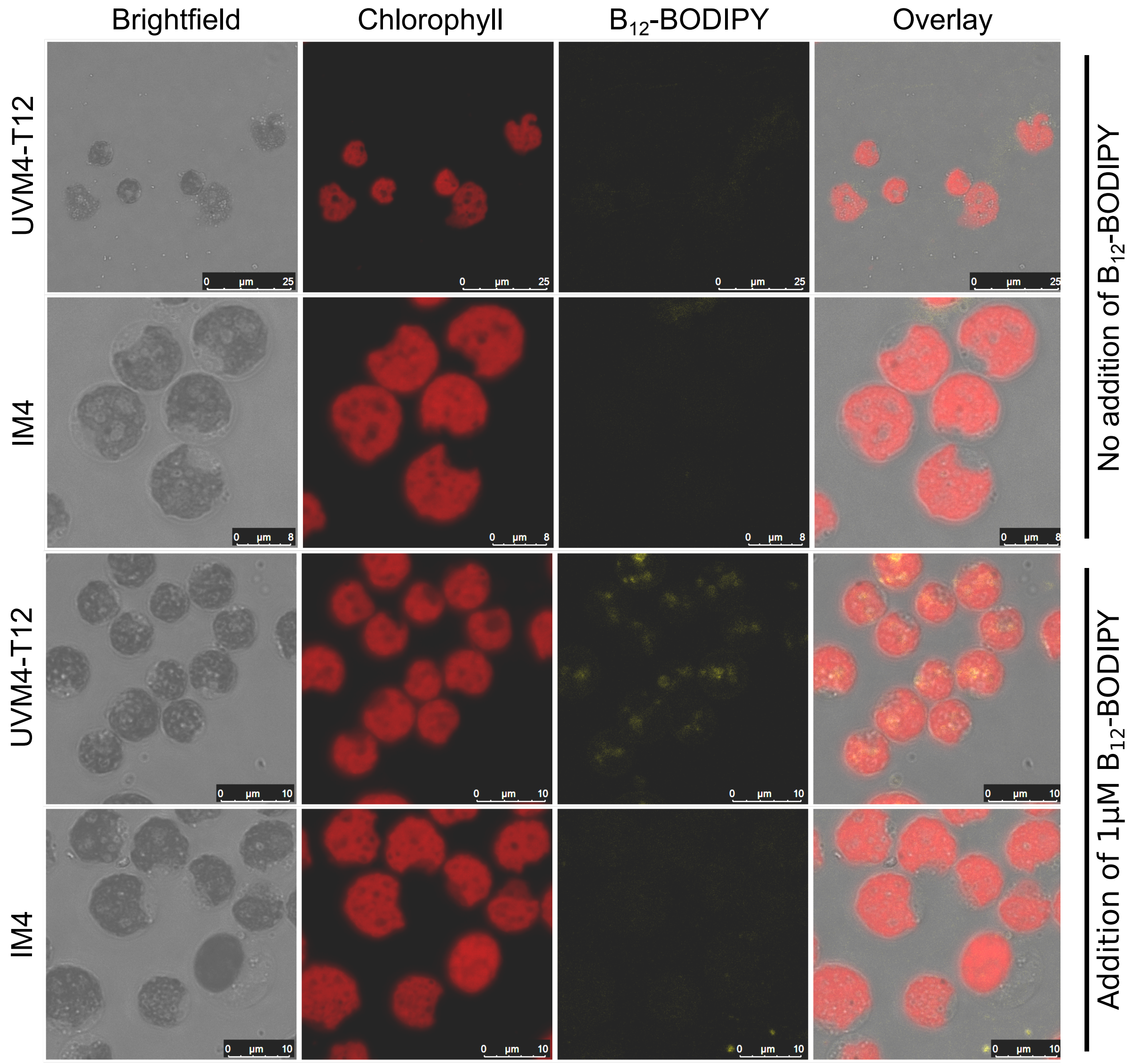

Figure S2. Visualization of B_{12}-BODIPY uptake in *C. reinhardtii* using confocal microscopy. To assess B_{12} uptake more directly, the insertional mutant line IM4 (impaired B_{12} uptake) and its parental line UVM4-T12 were incubated with the fluorescent B_{12} analogue B_{12}-BODIPY and the samples were imaged using confocal microscopy, as described in the materials and methods. Channels shown are brightfield (greyscale), chlorophyll (red), B_{12}-BODIPY (yellow) and an overlay.

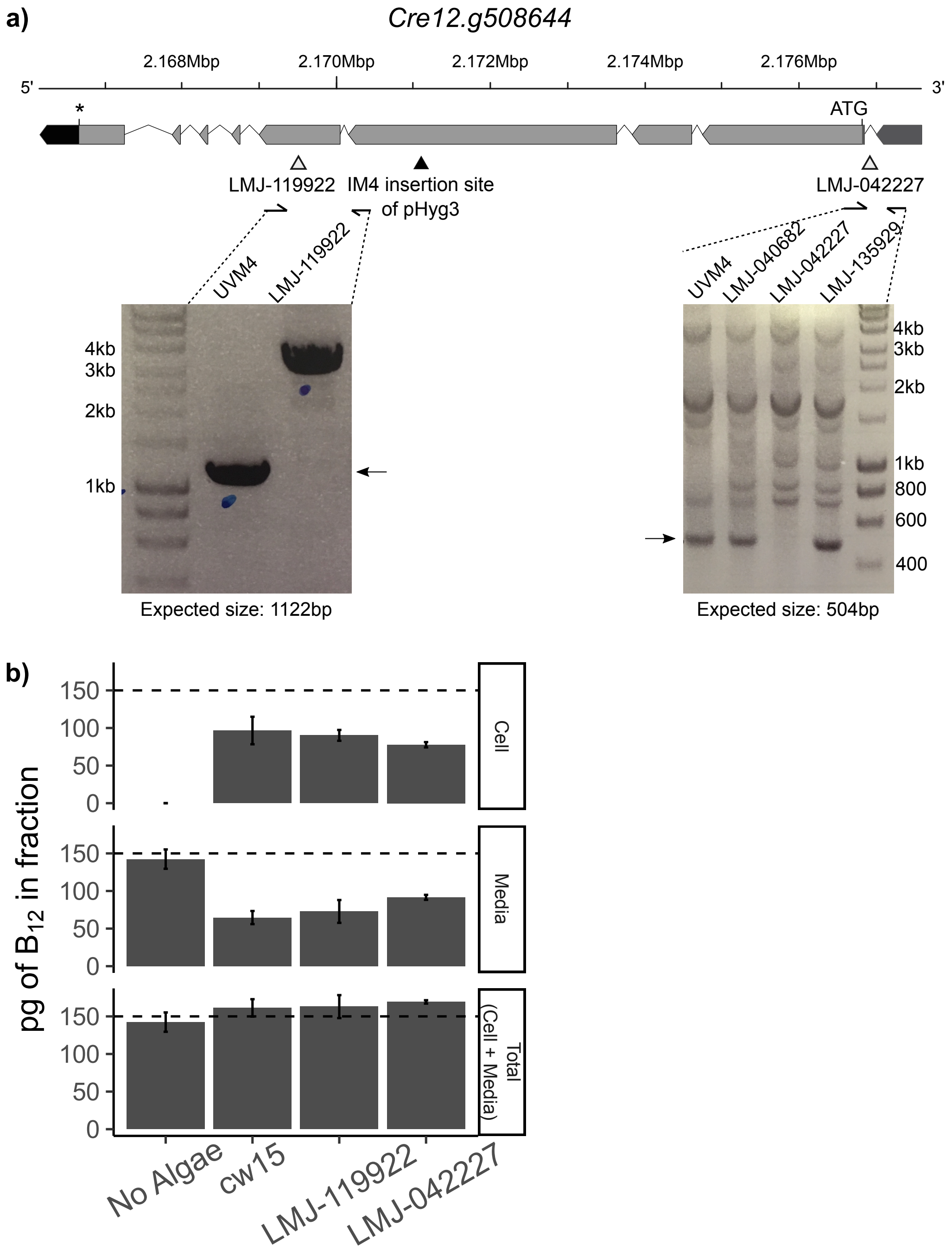

Figure S3. ***C. reinhardtii*** **knockout lines of** ***Cre12.g508644*** **are able to take up** $\mathbf{B_{12}}$**. a)** Schematic showing the structure of *Cre12.g508644* with annotations for the 5'UTR (medium grey), start codon (ATG), exons (light grey), introns (black lines), stop codon (*) and 3'UTR (black). The location of the IM4 pHyg3 insertion site (identified by DNA sequencing and validated using PCR) is indicated with a black triangle. The predicted disruption sites in CLiP (Li et al., 2016) knockout strains LMJ-119922 and LMJ-042227 are indicated with grey triangles. These were also confirmed by PCR (insets) **b)** B_{12}-uptake assay of the CLiP mutants and their background strain cw15. The dashed line shows the amount of B_{12} added to the experiment. Total = inferred by addition of amounts determined in the cellular and media fractions. Standard deviation error bars are shown, No Algae (n=10), cw15 (n=10), LMJ-119922 (n=10) and LMJ-042227 (n=6). Statistical analysis was performed on the media fraction, and Tukey's test identified the following comparisons to be significantly different from one another: No Algae vs cw15 ($p<1e^{-12}$); No Algae vs LMJ-119922 ($p<1e^{-12}$); No Algae vs LMJ-042227 ($p<1e^{-08}$); cw15 vs LMJ-042227 ($p<1e^{-03}$); and LMJ-119922 vs LMJ-042227 ($p<0.05$).

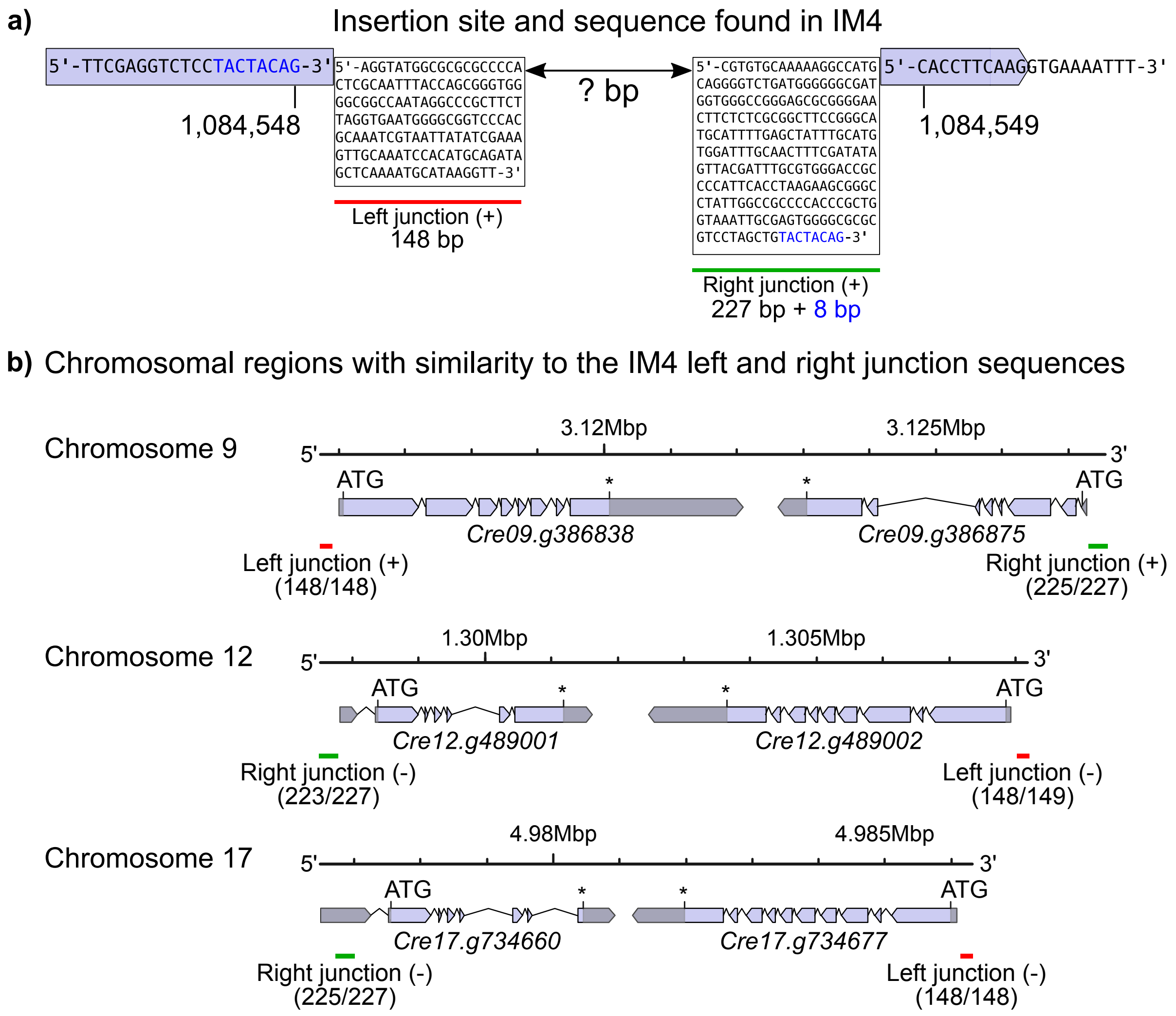

Figure S4. Structure and sequence of a second insertion in the IM4 strain. a) Mapping of the WGS data to the reference strain revealed an extra sequence between positions 1,084,548 bp and 1,084,549 bp of chromosome 2 in the *C. reinhardtii* v5.0 Phytozome reference genome corresponding to the Cre02.g081050 locus. An 8 bp target site duplication 'TACTACAG' was identified flanking the insertion (blue). The sequences of the left (red) and right (green) insertion junctions were identified from DNA sequencing reads and confirmed with PCR and sequencing. The sequence between these left and right junctions has not been determined. **b)** Chromosomal regions with similarity to the left (148 bp) and right (227 bp) junction sequences were identified by a BLAST search. Shown are three regions with sequence similarity to the left and right junctions and where the left and right junctions are a similar distance apart (~10 Kb) and in the same orientation. The position of the matching left junction (red) and right junction (green) sequences are indicated. The matching strand is indicated in brackets. The number of matching base pairs from the BLAST search is listed in brackets (target/query).

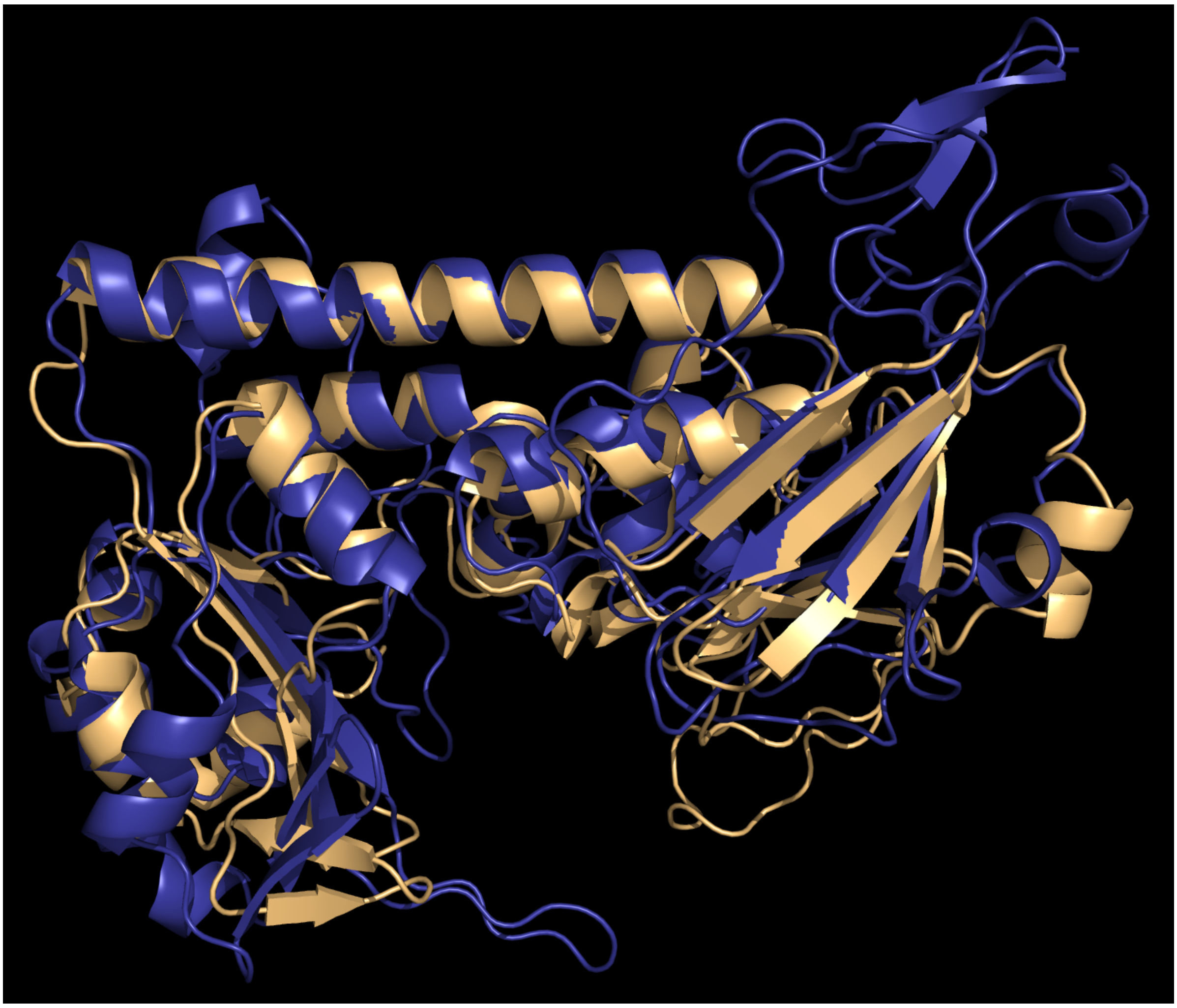

Figure S5. The predicted structures of CrCBA1 and PtCBA1 show a high degree of structural similarity. Structural predictions of CrCBA1 (Cre02.g081050) (blue) (residues 21-490) and PtCBA1 (Phatr3_J48322) (gold) (residues 51-370) were obtained from the AlphaFold Protein Database. These regions had a per-residue confidence score (pLDDT) of >70%, whereas residues at the N- and C-termini had much lower prediction confidence and so are omitted. The two structures were aligned using the super command in Pymol, root mean squared deviation = 1.688 between 1428 atoms. Conserved alpha helices are seen in the centre of the image, as is a common cleft region at the bottom of the image.

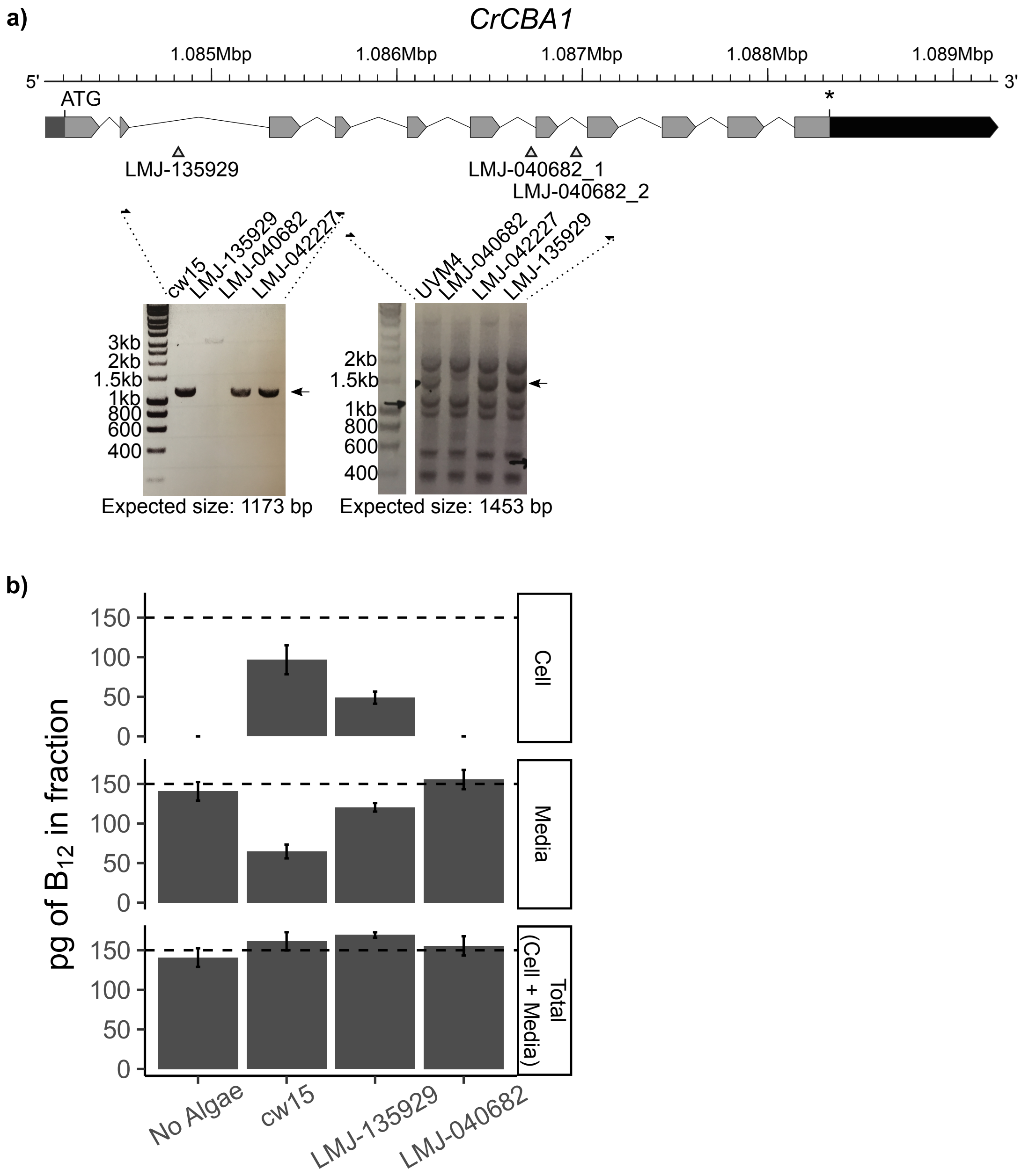

Figure S6. Independent mutant lines of *CrCBA1* show defective B_{12} uptake. A) Schematic showing the structure of *CrCBA1* (Cre02.g081050) with annotations for the 5'UTR (medium grey), start codon (ATG), exons (light grey), introns (black lines), stop codon (*) and 3'UTR (black). The location of predicted disruption sites in *CrCBA1* CliP knockout strains LMJ-135929 and LMJ-040682 are indicated (triangles). Primer positions used for the analysis of mutant lines are shown with arrows. Insets show that primers amplifying between exons 2 and 4 produced a band of the expected size for control lines cw15, LMJ-040682 and LMJ-042227, whereas LMJ-135929 showed an increased amplicon size, indicating an insertion in this region. Primers between exons 4 and 8 produced a band of the expected size in UVM4 and the same-background control lines LMJ-042227 and LMJ-135929, whereas LMJ-040682 lacked this band, indicating a disruption in this region. **B)** B_{12}-uptake assay. The dashed line indicates the amount of B_{12} added to the sample. Standard deviation error bars are shown, No Algae (n=14), cw15 (n=10), LMJ-135929 (n=6) and LMJ-040682 (n=18). Statistical analysis was performed on the media fraction, and Tukey's test identified the following comparisons to be significantly different from one another: No Algae vs cw15 ($p<1e^{-12}$); No Algae vs LMJ-135929 ($p<1e^{-02}$); No Algae vs LMJ-040682 ($p<1e^{-02}$); cw15 vs LMJ-135929 ($p<1e^{-11}$); cw15 vs LMJ-040682 ($p<1e^{-12}$); and LMJ-135929 vs LMJ-040682 ($p<1e^{-06}$).

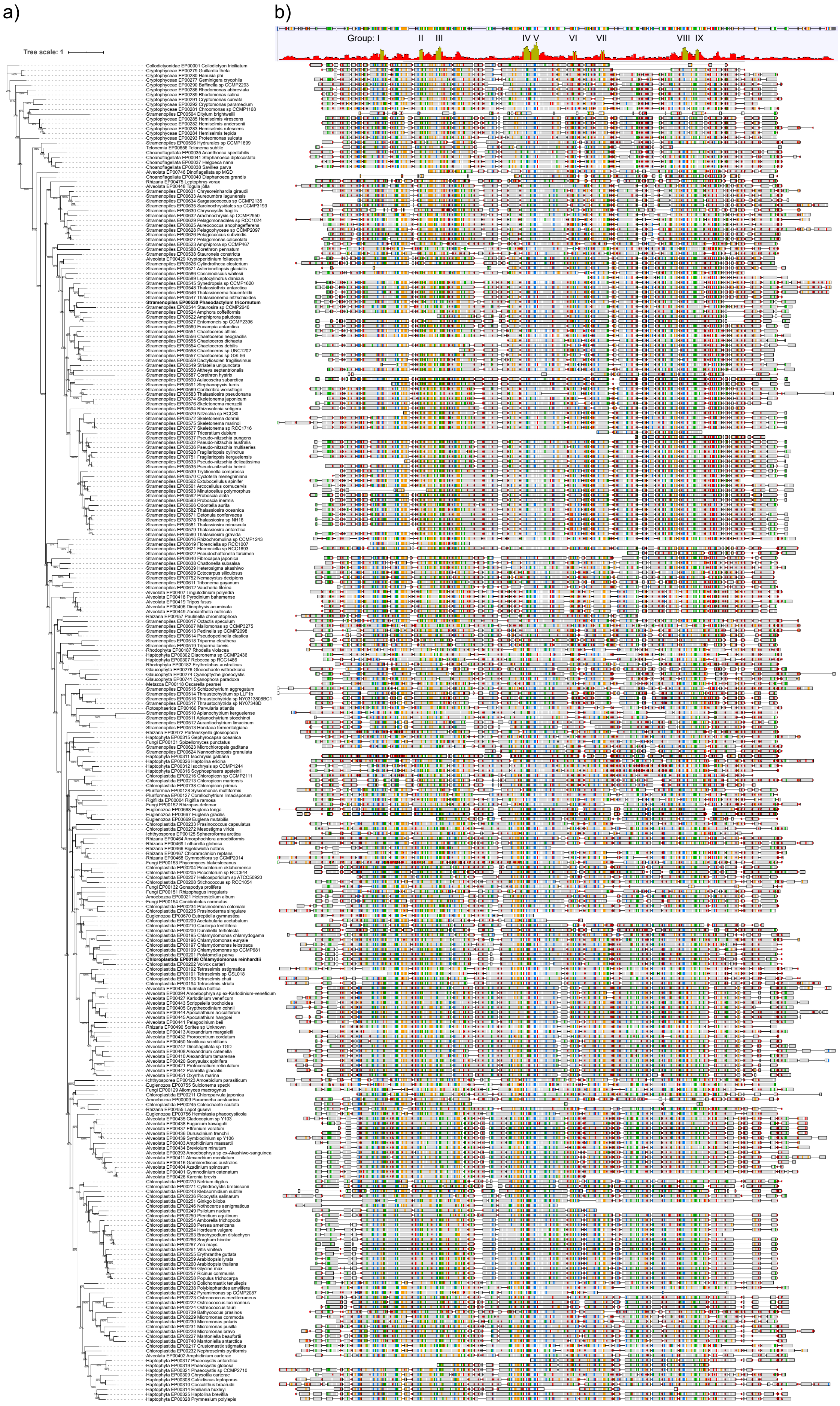
a)
b)
Tree scale: 1
Group: I
II
III
IV V
VI
VII
VIII IX

Figure S7. Sequences with similarity to CBA1 are found throughout Eukaryota. Sequences with similarity to CBA1 were identified from the EukProt database (Richter et al., 2022) using a manually generated hidden Markov model for CBA1, as described in the materials and methods. Positively classified CBA1 sequences were aligned with MAFFT (--auto option) (Katoh and Standley, 2013) and trimmed with trimai (-automated1 option) (Capella-Gutiérrez et al., 2009), and Iqtree version 1.6.10 (options: -bb 1000, -safe, -bnni, -alrt 1000, -st AA, -seed 1000, -msub nuclear, -t RANDOM, -m TEST) (Nguyen et al., 2015) was used to produce a gene tree. Visualization was performed using iTOL (Letunic and Bork, 2019). A multiple sequence alignment of the full length protein sequences was generated and visualized using Geneious Prime 2021.1.1 (https://www.geneious.com), and is shown adjacent to the tree (options: Clustal colour scheme, agreements to consensus highlighted, sliding window size = 5 bp, and sites with over 50% gaps hidden). A conservation track and consensus track are shown above the alignment panel, with regions of conserved residues indicated, detailed more in Figure 8a.

Supplemental Table S1 - Table of strains used in this work

Where no reference is listed the strain was obtained from the Culture Collection of Algae and Protozoa (CCAP), Oban, Scotland

Species	Type of Growth Media	Reference	Conditions
Phaeodactylum tricornutum CCAP 1055/1	F/2 media (Guillard, 1975)		18ºC, light intensity of 50 µE·m^{-2}·s^{-1} under a 16 h light / 8 h dark cycle and constant shaking at 110 rpm
Phaeodactylum tricornutum CCAP 1055/1 ΔCBA1-1; ΔCBA1-2; ΔCBA1-3;	F/2 media (Guillard, 1975) with nourseothricin or zeocin		18ºC, light intensity of 50 µE·m^{-2}·s^{-1} under a 16 h light / 8 h dark cycle and constant shaking at 110 rpm
Salmonella typhimurium AR3612 *cysG metE*	M9 Minimal medium with methionine (50 mg/L) and cysteine (50 mg/L)	Raux et al., 1996	37°C, shaking speed 220 rpm
Chlamydomonas reinhardtii UVM4	Tris-acetate phosphate (TAP) medium with Kropat's trace elements (Kropat et al., 2011) but excluding selenium	Neupert et al., 2009	25°C under a continuous or 16 h / 8 h light / dark cycle (shaking speed of 120 rpm, light intensity of 90 µmol.m^{-2}.s^{-1})
Chlamydomonas reinhardtii UVM4::pAS_R1 (UVM4-T12)	Tris-acetate phosphate (TAP) medium with Kropat's trace elements (Kropat et al., 2011) but excluding selenium. Paromomycin 5-50 µg/ml.	This work	25°C under a continuous or 16 h / 8 h light / dark cycle (shaking speed of 120 rpm, light intensity of 90 µmol.m^{-2}.s^{-1})
Chlamydomonas reinhardtii UVM4-T12::pHyg3 #IM1-7	Tris-acetate phosphate (TAP) medium with Kropat's trace elements (Kropat et al., 2011) but excluding selenium. Paromomycin 5-20 µg/ml, hygromycin 10-20 µg/ml.	This work	25°C under a continuous or 16 h / 8 h light / dark cycle (shaking speed of 120 rpm, light intensity of 90 µmol.m^{-2}.s^{-1})
Chlamydomonas reinhardtii CC-5325 cw15 mt- (cw15)	Tris-acetate phosphate (TAP) medium with Kropat's trace elements (Kropat et al., 2011) but excluding selenium.	Li et al., 2016	25°C under a continuous or 16 h / 8 h light / dark cycle (shaking speed of 120 rpm, light intensity of 90 µmol.m^{-2}.s^{-1})
Chlamydomonas reinhardtii LMJ-119922 and LMJ-042227	Tris-acetate phosphate (TAP) medium with Kropat's trace elements (Kropat et al., 2011) but excluding selenium. Paromomycin 5-20 µg/ml	Li et al., 2016	25°C under a continuous or 16 h / 8 h light / dark cycle (shaking speed of 120 rpm, light intensity of 90 µmol.m^{-2}.s^{-1})
IM4::pAS_C2	Tris-acetate phosphate (TAP) medium with Kropat's trace elements (Kropat et al., 2011) but excluding selenium. Paromomycin 5-20 µg/ml, hygromycin 10-20 µg/ml, Spectinomycin 75 µg/ml.	This work	25°C under a continuous or 16 h / 8 h light / dark cycle (shaking speed of 120 rpm, light intensity of 90 µmol.m^{-2}.s^{-1})
Chlamydomonas reinhardtii LMJ-040682 and LMJ-135929	Tris-acetate phosphate (TAP) medium with Kropat's trace elements (Kropat et al., 2011) but excluding selenium. Paromomycin 5-20 µg/ml	Li et al., 2016	25°C under a continuous or 16 h / 8 h light / dark cycle (shaking speed of 120 rpm, light intensity of 90 µmol.m^{-2}.s^{-1})
LMJ-040682::pAS_C2	Tris-acetate phosphate (TAP) medium with Kropat's trace elements (Kropat et al., 2011) but excluding selenium. Paromomycin 5-20 µg/ml, Spectinomycin 75 µg/ml, +/- 10 µM thiamine.	This work	25°C under a continuous or 16 h / 8 h light / dark cycle (shaking speed of 120 rpm, light intensity of 90 µmol.m^{-2}.s^{-1})
LMJ-040682::pAS_C3	Tris-acetate phosphate (TAP) medium with Kropat's trace elements (Kropat et al., 2011) but excluding selenium. Paromomycin 5-20 µg/ml, Spectinomycin 75 µg/ml +/- 10 µM thiamine.	This work	25°C under a continuous or 16 h / 8 h light / dark cycle (shaking speed of 120 rpm, light intensity of 90 µmol.m^{-2}.s^{-1})
WT12-metE7 mutant	Tris-acetate phosphate (TAP) medium with Kropat's trace elements (Kropat et al., 2011) but excluding selenium	Helliwell et al 2015	25°C under a continuous or 16 h / 8 h light / dark cycle (shaking speed of 120 rpm, light intensity of 90 µmol.m^{-2}.s^{-1})

Supplemental Table S2 - Plasmids used in this study.

The majority of plasmids were assembled following a Golden Gate cloning strategy and using previously developed parts (pCM0, Crozet et al. 2018) and backbone plasmids (pICH, Engler et al. 2014).

Name	Purpose	Description	Reference
pICH47742:PtFCP:Cas9YFP	*P. tricornutum* CRISPR-Cas9	Level 1 - Cas9-YFP, used in pMLP2117	Hopes et al. 2017
pCR8/GW:PtU6	*P. tricornutum* CRISPR-Cas9	Level 0 - PtU6 promoter used in pMLP2117	Hopes et al. 2017
pICH86966::AtU6p::sgRNA_PDS	*P. tricornutum* CRISPR-Cas9	Plasmid to amplify the sgRNA scaffold used in pMLP2117	Hopes et al. 2017
pMLP2117	*P. tricornutum* CRISPR-Cas9	Level 2 CRISPR-Cas9 construct,<br>containing Cas9-YFP (pICH47742:PtFCP:Cas9YFP), a zeocin resistance cassette and sgRNAs targeting *CBA1*	This study
pMLP2127	*P. tricornutum* CRISPR-Cas9	Homology template to create CBA1 KO mutants, Level 2 constructs, containing CBA1 flanking region, cloned on either side of a nourseothricin resistance cassette	This study
pAS_R1	B_{12} reporter	P_{METE}-*aphVIII*-T_{CA1}<br>Level 1 construct assembled using constructs pCM0-019, pCM0-074 and pCM0-117	This study
pHyg3	Insertional cassette	$P_{beta2\text{-}tubulin}$-*AphVII*-T_{Rbcs2},<br>Plasmid encodes hygromycin resistance cassette	Bertold et al. 2002
L0_P_Cre02g081050	Golden Gate, Complementation	Level 0 – Promoter and 5'UTR of *Cre02.g081050* cloned into pICH41295, used in L1_Cre02g081050-mVenus	This study
L0_CDSns_Cre02g081050	Golden Gate, Complementation	Level 0 – CDS of *Cre02.g081050* no stop codon cloned into pICH41258, used in L1_Cre02g081050-mVenus	This study
L0_T_Cre02g081050	Golden Gate, Complementation	Level 0 – 3'UTR and Terminator of *Cre02.g081050* cloned into pICH41276, used in L1_Cre02g081050-mVenus	This study
L0_CDS_Cre02g081050	Golden Gate, Complementation	Level 0 – CDS of *Cre02.g081050* cloned into pICH41308, used in L1_Cre02g081050-mVenus	This study
L1_SpecR	Golden Gate, Complementation	P_{PSAD}-*aada*-T_{PSAD}<br>Level 1 – Spectinomycin resistance cassette assembled using pCM0-016, pCM0-076 and pCM0-114, used in pAS_C2 and pAS_C3	This study
L1_Cre02g081050-mVenus	Golden Gate, Complementation	$P_{Cre02.g081050}$-*Cre02.g081050-mVenus*-$T_{Cre02.g081050}$<br>Level 1 – Expression cassette of *Cre02.g081050* fused to mVenus (pCM0-113), used in pAS_C2	This study
L1_RS-Cre02g081050	Golden Gate, Complementation	$P_{Rbcs2/Hsp70}$-*CrTHI4-4N-Cre02.g081050*-T_{CA1}<br>Level 1 - Expression cassette of *Cre02.g081050* under the control of the *CrTHI4-4N* riboswitch (pPM0-037, Mehrshahi et al. 2020), assembled with promoter pCM0-008 and terminator pCM0-117, used in pAS_C3	This study
pAS_C2	Complementation	P_{PSAD}-*aada*-T_{PSAD} $P_{Cre02.g081050}$-*Cre02.g081050-mVenus*-$T_{Cre02.g081050}$<br>Level 2 construct of L1_SpecR and L1_ Cre02g081050-mVenus	This study
pAS_C3	Complementation	P_{PSAD}-*aada*-T_{PSAD} $P_{Rbcs2/Hsp70}$-*CrTHI4-4N-Cre02.g081050*-T_{CA1},<br>Level 2 construct of L1_SpecR and L1_ RS-Cre02g081050	This study

Supplemental Table S3 - Oligonucleotides used in this study.
F/fwd and R/rv refer to forward and reverse primers, respectively. Restriction sites used for cloning are indicated in bold. Underlined nucleotides are specific 4nt overhangs used for Golden Gate-based cloning.

Name	Purpose	Sequence	Notes
sgCBA1.1.fwd	*P. tricornutum* cloning primer	AA**GGTCT**CACGAGGACCTACCTCCTCTACCA GTGGTTTTAGAGCTAGAAATAGCAAG	Used for pMLP2117
sgCBA1.2.fwd	*P. tricornutum* cloning primer	AA**GGTCT**CACGAGGCGTTTGCGAGAGACCCA CGTGTTTTAGAGCTAGAAATAGCAAG	Used for pMLP2117
sgRNA.rv	*P. tricornutum* cloning primer	T**GGTCT**CAAGCGTAATGCCAACTTTGTACAAG	Used for pMLP2117
CBA1.5HR.fwd	*P. tricornutum* cloning primer	AA**GGTCT**CAGGAGTCGACCACCACTTATTCC	Used for pMLP2127
CBA1.5HR.rv	*P. tricornutum* cloning primer	AA**GGTCT**CAAGCGTTCGGAGTATCCTGATGG	Used for pMLP2127
CBA1.3HR.fwd	*P. tricornutum* cloning primer	AA**GGTCT**CAGGAGGACTACTCAGTCTTATAC ACGTATTG	Used for pMLP2127
CBA1.3HR.rv	*P. tricornutum* cloning primer	AA**GGTCT**CAAGCGACACAGAAATGATGCCTC	Used for pMLP2127
gCBA1.fwd	*P. tricornutum* genotyping primer	GTTTCCCCCAAGCCTTTG	White triangle in Fig.1
gCBA1.rv	*P. tricornutum* genotyping primer	CAGCAAGGACGCTATTCAGG	White triangle in Fig.1
gCBA1in.fwd	*P. tricornutum* genotyping primer	CGCTCTTCTCCCAAGGATG	Grey arrow in Fig.1
gCBA1in.rv	*P. tricornutum* genotyping primer	GATCTCGTCCAAGAAGCAAGG	Grey arrow in Fig.1
NAT.rv	*P. tricornutum* genotyping primer	AGTGAACACGACGCTGAAGG	Grey arrow with circle in Fig.1
METE F	RT-qPCR METE	CCGCTACAGCCAGACTTCA	Fig. 3a
METE R	RT-qPCR METE	GTGACAGCGACACGAACGT	Fig. 3a
ON_56 F	RT-qPCR RACK1	CGTCTGTGGGACCTGAACAC	Fig. 3a
ON_57 R	RT-qPCR RACK1	GCTCGCCAATGGTGTACTTG	Fig. 3a
ON_135 F	LMJ-119922 knockout confirmation	CACCACCAGCACCCAGTG	Fig. S3a
ON_141 R	LMJ-119922 knockout confirmation	GCTCCCGAACCCGTCAC	Fig. S3a
ON_177 F	LMJ-042227 knockout confirmation	AGCAGCAGTAGAAGCAGCG	Fig. S3a
ON_178 R	LMJ-042227 knockout confirmation	GGCTGTACTCCGCCTCCAT	Fig. S3a
ON_121 F	*Golden Gate Cre02.g081050* promoter	TT**GAAGAC**ATGGAGCAGCAGTCGCTGTGTCTC GTACTCCAC	To assemble L0_P_Cre02g081050
ON_151 R	*Golden Gate Cre02.g081050* promoter	TT**GAAGAC**ATACACATTTGACACAATGTCGTT TCCCAAGTTTCAGGG	To assemble L0_P_Cre02g081050
ON_152 F	*Golden Gate Cre02.g081050* promoter	TT**GAAGAC**ATGTGTTCACGCCATGCGCC	To assemble L0_P_Cre02g081050
ON_153 R	*Golden Gate Cre02.g081050* promoter	TT**GAAGAC**ATGGTGTCGGGGGAGGCCTTG	To assemble L0_P_Cre02g081050
ON_154 F	*Golden Gate Cre02.g081050* promoter	TT**GAAGAC**ATCACCTCGTTCTCCTTGTCAGCC ACGCAATCGCAAGGTTGGATCTTC	To assemble L0_P_Cre02g081050
ON_155 R	*Golden Gate Cre02.g081050* promoter	TT**GAAGAC**ATGGTATCGCCACTACCGCAACCC AGTCCGC	To assemble L0_P_Cre02g081050
ON_156 F	*Golden Gate Cre02.g081050* promoter	TT**GAAGAC**ATTACCCCGTGCTCCTCGC	To assemble L0_P_Cre02g081050
ON_122 R	*Golden Gate Cre02.g081050* promoter	TT**GAAGAC**ATCATTCACAATGTATGTGTAGCG CAACCTTGC	To assemble L0_P_Cre02g081050
ON_123 F	*Golden Gate Cre02.g081050* terminator	TT**GAAGAC**ATGCTTGCGCCCCGCCTCCCAG	To assemble L0_T_Cre02g081050
ON_161 R	*Golden Gate Cre02.g081050* terminator	TT**GAAGAC**ATAGATCGCGGGACCACCGTCTA CC	To assemble L0_T_Cre02g081050
ON_162 F	*Golden Gate Cre02.g081050* terminator	TT**GAAGAC**ATATCTCATGACTAACTGAAAGGT GCGGCGTGG	To assemble L0_T_Cre02g081050
ON_124 R	*Golden Gate Cre02.g081050* terminator	TT**GAAGAC**ATAGCGAGCAATGTTCTATTGTTT GCGGGGGATTCGG	To assemble L0_T_Cre02g081050
ON_112 F	*Golden Gate Cre02.g081050* CDS	TT**GAAGAC**ATAATGGCGTCTCGCGGCCTCTT	To assemble L0_CDSns_Cre02g081050 and L0_CDS_Cre02g081050
ON_157 R	*Golden Gate Cre02.g018050* CDSns	TT**GAAGAC**ATACACATTCACGCCGGTGACCTT CAG	To assemble L0_CDSns_Cre02g081050

ON_158 F	*Golden Gate Cre02.g018050* CDSns	TT**GAAGAC**ATGTGTTCCCCACGACGCG	To assemble L0_CDSns_Cre02g081050
ON_159 R	*Golden Gate Cre02.g018050* CDSns	TT**GAAGAC**ATGATACCTCGAAGTTCTGAGCCAC	To assemble L0_CDSns_Cre02g081050
ON_160 F	*Golden Gate Cre02.g018050* CDSns / LMJ_135929 knockout confirmation	TT**GAAGAC**ATTATCCTACTACAGCACCTTCAAGGTGAAAATTTCAGAATGCC	To assemble L0_CDSns_Cre02g081050 / Fig. S6a
ON_113 R	*Golden Gate Cre02.g018050* CDSns / LMJ_135929 knockout confirmation	TT**GAAGAC**ATAGGGGTGACGTACGCCACGCG	To assemble L0_CDSns_Cre02g081050 / Fig. S6a
ON_114 F	*Golden Gate Cre02.g018050* CDSns / LMJ-040682 knockout confirmation	TT**GAAGAC**ATCCCTTACGCCGTGGAGCCTTGC	To assemble L0_CDSns_Cre02g081050 / Fig. S6a
ON_116 R	*Golden Gate Cre02.g018050* CDSns / LMJ-040682 knockout confirmation	TT**GAAGAC**ATGGTGCGCAGCACCGGCAG	To assemble L0_CDSns_Cre02g081050 / Fig. S6a
ON_118 F	*Golden Gate Cre02.g018050* CDSns	TT**GAAGAC**ATCACCATCCTGGCCAGCGTGGATGTGG	To assemble L0_CDSns_Cre02g081050
ON_120 R	*Golden Gate Cre02.g018050* CDSns	TT**GAAGAC**ATACCTCCCAGGCCCCAGCCCAGCACG	To assemble L0_CDSns_Cre02g081050
ON_119 R	*Golden Gate Cre02.g081050* CDS	TT**GAAGAC**ATAAGCTTACAGGCCCCAGCCCAGCACG	To assemble L0_CDS_Cre02g081050
ON_104 F	pHyg3 insertion confirmation	CCATCGCTGTCACTGGGT	*Cre12.g508644* across insertion, left junction
ON_106 R	pHyg3 insertion confirmation	GATGACGCAGACTTTGCCAC	*Cre12.g508644* across insertion
ON_89 R	pHyg3 insertion confirmation	TACGGTCGAGAAGTAACAGGGA	*Cre12.g508644* left junction
ON_79 F	pHyg3 insertion confirmation	AAAACCCTGGCGTTACCCAA	*Cre12.g508644* right junction
ON_106 R	pHyg3 insertion confirmation	GATGACGCAGACTTTGCCAC	*Cre12.g508644* right junction
ON_205 F	Cre02.g081050 disruption confirmation	CCTTGTCAGCCACGCAATC	*Cre02.g081050* left junction
ON_201 R	Cre02.g081050 disruption confirmation	GCTTCCGGGCGAGAACCTTATG	*Cre02.g081050* left junction
ON_208 R	Cre02.g081050 disruption confirmation	GCAAGCAGACGGAGGACAAC	*Cre02.g081050* right junction
ON_203 F	Cre02.g081050 disruption confirmation	CGTGTGCAAAGGGCCATG	*Cre02.g081050* right junction

Supplemental Methods

Design of constructs for CRISPR/Cas9 editing of P. tricornutum
Plasmid pMLP2117 (Table S2), featuring Cas9-YFP, two sgRNAs and a zeocin resistance cassette was constructed following the Golden Gate cloning approach. A homology template was designed that contained 2 homology regions of approximately 800 bp from regions flanking the *P. tricornutum CBA1* (*PtCBA1)* ORF (Figure 1a), which were amplified from the genome and placed either side of a nourseothricin resistance cassette in plasmid pMLP2127. The level 1 plasmid encoding the Cas9-YFP expression cassette (pICH47742:PtFCP:Cas9YFP), the level 0 plasmid containing the PtU6 promoter used to drive expression of the sgRNAs (pCR8/GW:PtU6) and the plasmid used as a template to amplify the sgRNA scaffold (pICH86966::AtU6p::sgRNA_PDS) were gifts from Dr. Amanda Hopes and Prof Thomas Mock (University of East Anglia) (Hopes et al., 2017) and are available on Addgene. All primer sequences used in the cloning process can be found in Supplemental Table S2.

Algal dose-response assay
The algal dose response assay used to screen and quantitatively assess UVM4::pAS_R1 transformant lines was performed in the following manner. Firstly, three cultures of each line were grown in 96-well microtiter plates containing 200 µl TAP media supplemented with different concentrations of paromomycin and vitamin B_{12} (Figure 2b); after four days, optical density was measured at 730 nm (OD730) using a FluoStar OPTIMA (BMG labtech) plate reader; and finally, the data generated was modelled using a 4 parameter logistic equation (Ritz et al., 2015).

B_{12}-BOIDIPY Imaging
C. reinhardtii strains were incubated with 1 µM B_{12}-BODIPY for 1 hour at room temperature. Cells were pelleted by centrifugation at 5000 g and washed with TAP media 3 times. *C. reinhardtii* strains were imaged in a confocal laser scanning microscope (TCS SP8, Leica Microsystems, Germany) with an HC PL APO CS2 40x/1.30 aperture oil-immersion lens. Images were taken using the sequential mode provided by the Leica LAS software, with the channel used for chlorophyll and brightfield detection being taken first and the channel used for B_{12}-BODIPY detection taken second. The first image was acquired with excitation from a white light source at 476 nm at 6% power and emissions were detected between 674 - 688 nm; chlorophyll settings included 10% gain. Brightfield imaging used 699.1% gain and a 2.89% offset. Frames were captured with a line average of 16 and a frame accumulation of 1. The second image was acquired with excitation from a white light source at 589 nm at 2% power and emissions were detected between 607 - 620 nm with 500% gain. Frames were captured with a line average of 6 and a frame accumulation of 4. The overlay images were produced automatically by the Leica LAS software. Inkscape was used to increase the lightness and contrast of all the images in the same manner.

Identification of TE insert
Standard BLAST searches against the *C. reinhardtii* reference genome CC-503 v5 were performed using the BLASTN Tool integrated into the Phytozome database, the Plant Comparative Genomics portal of the Department of Energy's Joint Genome Institute (https://phytozome.jgi.doe.gov/pz/portal.html#). As comparison matrix BLOSUM62 was used with a default word length of 11 bp. In addition, gaps were allowed and filter query settings were set on. Multiple sequence alignments (MSA) were generated employing the progressive aligner MUSCLE (Edgar, 2004) which featured rapid sequence distance estimation using k-mer counting, implemented as part of the UGENE bioinformatic suite (v38.1, Okonechnikov et al., 2012). Default parameters, optimized for best accuracy were used. Consensus sequences from nucleotide or peptide alignments were extracted using UGENE's default consensus mode. BLAST searches against the NCBI nucleotide collection and non-redundant protein archive were performed using the MEGABLAST and BLASTP tools provided via the NIH website (https://blast.ncbi.nlm.nih.gov/Blast.cgi) using default paraments. Analysis of protein sequences for Pfam matches were conducted using the sequence search function from the Pfam server (http://pfam.xfam.org) applying default settings. The Repbase TE library for *C. reinhardtii* and relatives (https://www.girinst.org/repbase/) as well as the manually curated repeat

library generated by Craig et al. (2021) (repeat_lib_v3_2.volvocales) were used to annotated the identified repetitive element.

Supplemental references

Berthold P, Schmitt R, Mages W (2002). An engineered *Streptomyces hygroscopicus* aph 7" gene mediates dominant resistance against hygromycin B in *Chlamydomonas reinhardtii* . Protist: **153**(4):401-12.

Capella-Gutiérrez S, Silla-Martínez Joem, Gabaldón T (2009) trimAl: A tool for automated alignment trimming in large-scale phylogenetic analyses. Bioinformatics **25**: 1972–1973

Craig RJ, Hasan AR, Ness RW, Keightley PD (2021) Comparative genomics of *Chlamydomonas*. Plant Cell **33**:1016-1041

Crozet P, Navarro FJ, Willmund F, Mehrshahi P, Bakowski K, Lauersen KJ, Pérez-Pérez M-E, Auroy P, Gorchs Rovira A, Sauret-Gueto S, et al (2018) Birth of a photosynthetic chassis: A MoClo toolkit enabling synthetic biology in the microalga *Chlamydomonas reinhardtii*. ACS Synthetic Biology **7**: 2074–2086

Edgar RC (2004) MUSCLE: multiple sequence alignment with high accuracy and high throughput. Nucleic Acids Res 32, 1792–1797 (2004).

Engler C, Youles M, Gruetzner R, Ehnert TM, Werner S, Jones JD, Parton NJ, Marillonnet S (2014). A golden gate modular cloning toolbox for plants. ACS Synthetic Biology **3**: 839–843.

Guillard RRL (1975) Culture of phytoplankton for feeding marine invertebrates. *In* WL Smith, MH Chanley, eds, Culture of marine invertebrate animals: Proceedings —1st conference on culture of marine invertebrate animals greenport. Springer US, Boston, MA, pp 29–60

Hopes A, Nekrasov V, Belshaw N, Grouneva I, Kamoun S, Mock T (2017). Genome Editing in Diatoms Using CRISPR-Cas to Induce Precise Bi-allelic Deletions. Bio-Protocol **7**: 23

Katoh K, Standley DM (2013) MAFFT multiple sequence alignment software version 7: Improvements in performance and usability. Molecular Biology and Evolution **30**: 772–780

Kelley LA, Mezulis S, Yates CM, Wass MN, Sternberg MJE (2015) The Phyre2 web portal for protein modeling, prediction and analysis. Nat Protoc **10**: 845–58

Letunic I, Bork P (2019) Interactive tree of life (iTOL) v4: Recent updates and new developments. Nucleic Acids Research **47**: W256–W259

Mehrshahi P, Nguyen GTDT, Gorchs Rovira A, Sayer A, Llavero-Pasquina M, Lim Huei Sin M, Medcalf EJ, Mendoza-Ochoa GI, Scaife MA, Smith AG (2020) Development of novel riboswitches for synthetic biology in the green alga *Chlamydomonas*. ACS Synthetic Biology **9**: 1406–1417

Nguyen L-T, Schmidt HA, Haeseler A von, Minh BQ (2015) IQ-TREE: A fast and effective stochastic algorithm for estimating maximum-likelihood phylogenies. Molecular Biology and Evolution **32**: 268–274

Okonechnikov K, Golosova O, Fursov M, Team U (2012) Unipro UGENE: a unified bioinformatics toolkit. Bioinformatics 28, 1166–1167

Raux-Deery E, Lanois A, Levillayer F, Warren MJ, Brody E, Rambach A, Thermes C (1996) *Salmonella typhimurium* cobalamin (vitamin B_{12}) biosynthetic genes: Functional studies in *S. typhimurium* and *Escherichia coli.* Journal of Bacteriology **178**: 753–67

Ritz C, Baty F, Streibig JC, Gerhard D (2015) Dose-response analysis using R. PLoS One **10**: e0146021
